# Detection of Cervical Nodal Metastasis in Oral Squamous Cell Carcinoma Using Elastic Scattering Spectroscopy: Ex Vivo Diagnostic Modeling Study

**DOI:** 10.2196/103960

**Published:** 2026-10-13

**Authors:** Waseem Jerjes, Zaid Hamdoon, Dara Rashed, Colin Hopper

**Affiliations:** 1 Department of Primary Care and Public Health, Imperial College London, 90 Wood Lane, London, W12 0BZ, United Kingdom, 44 07471479868; 2 College of Dental Medicine, University of Sharjah, Sharjah, United Arab Emirates; 3 Unit of Oral and Maxillofacial Surgery, UCL Eastman Dental Institute, London, United Kingdom

**Keywords:** oral squamous cell carcinoma, cervical lymph node metastasis, elastic scattering spectroscopy, optical biopsy, diagnostic accuracy, decision support systems, optical diagnostics, head and neck cancer, ex vivo tissue assessment

## Abstract

**Background:**

Cervical nodal metastasis remains one of the strongest determinants of treatment planning and prognosis in oral squamous cell carcinoma. Histopathology remains the reference standard, but it does not provide immediate information at the point of surgical specimen assessment. Elastic scattering spectroscopy (ESS) offers rapid, label-free optical interrogation of tissue microstructure, and may support ex vivo nodal assessment.

**Objective:**

This study aimed to evaluate the diagnostic accuracy of ESS combined with machine learning classification for ex vivo detection of cervical lymph node metastasis in patients with oral squamous cell carcinoma.

**Methods:**

We conducted a single-center observational ex vivo diagnostic modeling study in patients with oral squamous cell carcinoma undergoing primary tumor resection and neck dissection. A total of 3 ESS spectra were acquired from each bivalved lymph node before routine histopathological processing. Internal validation used repeated nested 5-fold cross-validation grouped by patient, so every spectrum and lymph node from a patient remained in the same fold. All preprocessing, scaling, dimensionality reduction, within-model-family configuration, and hyperparameter tuning, probability calibration where required, and decision-threshold selection were restricted to training data within the nested validation process; comparisons between candidate model families were exploratory and were based on pooled patient-grouped out-of-fold predictions. Spectrum-level probabilities were aggregated within each lymph node, and histopathology was the reference standard.

**Results:**

A total of 52 patients were assessed for eligibility, 45 were included, and 280 fresh lymph nodes were assessed by ESS; 96 were pathology positive, and 184 were pathology negative. Of 840 acquired spectra, 760 passed quality control. Of the 280 nodes, 274 retained at least 2 usable spectra and comprised the analytical cohort; 6 pathology-negative nodes yielded no analyzable node-level ESS result. The hybrid model was selected because it had the highest pooled patient-grouped out-of-fold node-level area under the receiver operating characteristic curve (AUROC), with an internally validated, exploratory estimate of 0.966 (95% CI 0.929‐0.988). The same out-of-fold predictions informed model-family selection and performance reporting; this estimate does not represent independent validation of a prospectively fixed final model. At the cross-validation–derived summary threshold, secondary descriptive operating characteristics were sensitivity 93.8% (95% CI 86.4%‐97.6%), specificity 95.5% (95% CI 90.1%‐98.3%), accuracy 94.9% (95% CI 90.4%‐97.3%), positive predictive value 91.8% (95% CI 82.9%‐96.3%), and negative predictive value 96.6% (95% CI 92.4%‐98.8%). The Brier score was 0.063, with calibration-in-the-large of –0.03 and a calibration slope of 0.94. Patient-level sensitivity was 96% and specificity was 90%. Pathology-positive and pathology-negative nodes also showed differences across amplitude-related and shape-related spectral features.

**Conclusions:**

ESS showed promising ex vivo discrimination of metastatic cervical lymph nodes in this single-center model-development and internal-validation study. The findings support further investigation of ESS as a potential pathology-triage adjunct rather than a replacement for histopathology.

## Introduction

Head and neck squamous cell carcinoma remains a major global cancer burden, and oral squamous cell carcinoma is one of its most clinically demanding subtypes because small differences in regional disease can substantially alter treatment. Contemporary global estimates confirm the scale of cancer incidence and mortality, while epidemiological reviews continue to identify tobacco, alcohol, areca nut, human papillomavirus infection, and social determinants as important contributors to disease distribution and late presentation [1,2]. For the surgeon and oncologist, however, the decisive issue at the time of treatment planning is often not the primary tumor alone, but whether malignant disease has reached the cervical lymphatic system. Once cervical nodal metastasis is identified, staging, operative planning, adjuvant radiotherapy or chemoradiotherapy, surveillance intensity, and prognosis all change [3].

The clinical importance of the neck in oral cancer is reflected in the depth of work examining predictors and patterns of nodal metastasis. Recent systematic reviews show that histopathological features of the primary tumor, including depth of invasion and adverse growth patterns, are associated with cervical lymph node metastasis, and that nodal involvement varies across neck levels in ways that influence decisions about the extent of neck dissection [4,5]. Yet the fundamental problem remains unresolved—occult or small-volume nodal metastases may be difficult to identify before treatment, while enlarged or reactive nodes may be mistaken for malignant disease. The consequence is a familiar clinical tension between undertreatment of metastatic disease and overtreatment of patients whose necks are ultimately histologically negative.

Preoperative imaging has improved this balance but has not eliminated uncertainty. Computed tomography (CT), magnetic resonance imaging (MRI), positron emission tomography-computed tomography (PET-CT), ultrasound, and radiomics-based approaches provide valuable information, and PET-CT may offer high specificity and negative predictive value (NPV) in selected clinically node-negative settings [6,7]. However, meta-analyses and radiomics reviews also highlight variable sensitivity, dependence on tumor site and study design, and a continuing need for prospective validation before advanced computational imaging can be relied upon for occult nodal disease [6-8]. Imaging therefore remains essential, but it cannot replace detailed histopathological examination of dissected lymph nodes. Equally, histopathology is accurate but not immediate. Routine processing, sectioning, and reporting take time, and the workload generated by multiple lymph nodes can be considerable.

A second challenge is that nodal assessment is intrinsically heterogeneous. A neck dissection specimen may contain many nodes of different sizes and anatomical levels, and metastatic deposits may be focal, central, peripheral, or microscopic. Standard histopathology is designed to manage this complexity, but the process depends on careful grossing, representative sectioning, and prioritization of tissue that appears suspicious. A rapid adjunctive technology would therefore be valuable if it could identify nodes with a high probability of metastasis before or during routine processing. Such a test would not need to provide the final diagnosis to be useful; it would need to improve where attention is directed, how quickly suspicious nodes are reviewed, and how confidently low-risk nodes are handled within established pathology protocols.

Optical diagnostic technologies have been developed to address exactly this gap; the need for rapid, biologically meaningful information from tissue without waiting for full laboratory processing. Recent reviews of oral cancer diagnostics and head and neck photodiagnosis describe several label-free or minimally invasive modalities, including Raman spectroscopy, fluorescence, hyperspectral imaging, and infrared spectroscopy combined with machine learning [9-13]. These techniques exploit changes in tissue architecture, scattering, absorption, and molecular composition that accompany dysplasia, invasion, and metastatic transformation. Their appeal in head and neck oncology is not that they replace histopathology, but that they may provide a near-immediate adjunct to help guide sampling, prioritize suspicious tissue, and improve the efficiency of diagnostic pathways. In lymph nodes, such optical signals may reflect not only the presence of malignant cells themselves but also the architectural consequences of metastatic replacement, including altered cellular density, stromal organization, vascularity, and loss of normal nodal microstructure.

Elastic scattering spectroscopy (ESS) is particularly suited to this role because it is rapid, contact-based, and sensitive to cellular and subcellular structure. When light enters tissue, it may be scattered or absorbed depending on wavelength, tissue architecture, nuclear size, stromal organization, and the distribution of chromophores. Earlier work in oral cancer showed that ESS could detect cervical intranodal metastasis in bivalved lymph nodes using intensity differences at selected wavelengths, although specificity was limited when a simple threshold was used [14]. Broader optical biopsy literature has also shown that elastic scattering can provide a strong optical signal in several epithelial and nodal settings, supporting the concept that tissue microstructure can be converted into clinically useful diagnostic information [15,16].

This study builds on that foundation by treating nodal assessment as a node-level diagnostic problem rather than a single-contact spectral problem. We hypothesized that metastatic lymph nodes would show reproducible amplitude-related and shape-related spectral differences and that combining full-spectrum information with selected optical features might improve discrimination compared with a simple single-wavelength rule. Because modern diagnostic algorithms require more than high apparent accuracy, we also designed the analysis to reflect principles of transparent prediction model reporting, diagnostic accuracy reporting, bias assessment, and early clinical evaluation of decision support systems [17-20]. The aim was to evaluate whether ESS could provide accurate ex vivo node-level discrimination of metastatic cervical nodal disease in oral squamous cell carcinoma and to define a clinically interpretable workflow for future validation.

## Methods

### Study Design, Setting, and Timeline

This observational ex vivo diagnostic modeling study was conducted at University College London Hospitals. The optical-imaging study received a favorable ethical opinion from The Joint UCL/UCLH Committees on the Ethics of Human Research (Committee A), confirmed in a letter dated July 31, 2006 (REC [Research Ethics Committee] reference 06/Q0505/39). Patients underwent primary tumor resection and neck dissection between September 2016 and March 2021, with fresh lymph-node preparation, ESS acquisition, and routine histopathological assessment undertaken during the same surgical episode. The archived spectra and pathology records were curated between January and December 2025, and the modeling analyses were completed between January and May 2026.

### Participants and Specimens

Potentially eligible patients were assessed during the recruitment period. Adults with histologically confirmed oral squamous cell carcinoma were eligible when neck dissection formed part of their planned surgery, fresh cervical lymph nodes were available before fixation, and written informed consent had been obtained. Patients were excluded when consent was not obtained, suitable fresh lymph nodes were unavailable before fixation, or the ESS system was unavailable at the time of surgery. The patient was the enrollment and validation unit; lymph nodes were clustered within patients, and repeated spectra were clustered within lymph nodes. Within each enrolled neck dissection, ESS was performed on fresh lymph nodes that could be isolated and made available before fixation without delaying or compromising routine pathological processing. The ESS-assessed nodes therefore represented a pragmatic availability-based sample and did not necessarily constitute the complete pathological nodal yield from each neck dissection. Selection for ESS assessment occurred before the final histopathological diagnosis was known.

### Ethical Considerations

The application for the optical-imaging study, “The Role of Optical Imaging (Elastic Scattering Spectroscopy, Raman Spectroscopy and Second-harmonic Imaging Microscopy) as a Diagnostic Tool in Head and Neck Cancer,” was submitted to The Joint UCL/UCLH Committees on the Ethics of Human Research (Committee A) on April 26, 2006. A favorable ethical opinion was confirmed in a letter dated July 31, 2006 (REC reference 06/Q0505/39), following consideration of further information by the Chair on July 28, 2006. The approved documents included protocol version 1.4, dated March 13, 2006; participant information sheet version 2, dated June 12, 2006; and participant consent form version 1.

The study protocol described comparison of optical imaging techniques with routine histopathology using clinically obtained oral or head and neck tissue and eligible archived material. The protocol described in vivo assessment before routine biopsy and ex vivo assessment of clinically removed oral or head and neck tissue, without additional research-only biopsy or surgery or interference with routine histopathological processing. The study continuation was subject to annual local organizational governance checks. The protocol assigned responsibility for study conduct, protocol compliance, data custody, and reporting to the Chief Investigator, who is a coauthor of this report.

This report concerns fresh cervical lymph-node ESS measurements acquired between September 2016 and March 2021. The archived spectra and pathology records were curated between January and December 2025, and modeling was undertaken between January and May 2026. All participants provided written informed consent for study participation. The analytical dataset was deidentified before modeling, direct identifiers were removed, and access was restricted to the study team. Participants received no payment or other compensation.

### Procedure and ESS System

After surgical removal, the neck dissection specimen was orientated with pathological guidance to preserve anatomical context and nodal identity. Individual lymph nodes were isolated and bivalved to expose the internal cut surface. Two trained operators performed ESS acquisition according to a written protocol, with each lymph node assessed by 1 operator. The freshly cut surface was gently blotted, visibly blood-contaminated areas were avoided, and the probe was held approximately perpendicular to the surface in gentle contact without visible tissue compression. In total, 3 spectra were acquired from spatially separated points across the exposed cut surface of each bivalved lymph node. Operator, acquisition batch, and the times of excision, bivalving, and first ESS acquisition were recorded. The median interval from excision to the first acquisition was 18 (IQR 13‐26) minutes. The overall diagnostic pathway is illustrated in Figure 1, and the probe-tissue interrogation geometry is illustrated in Figure 2.

**Figure 1.**
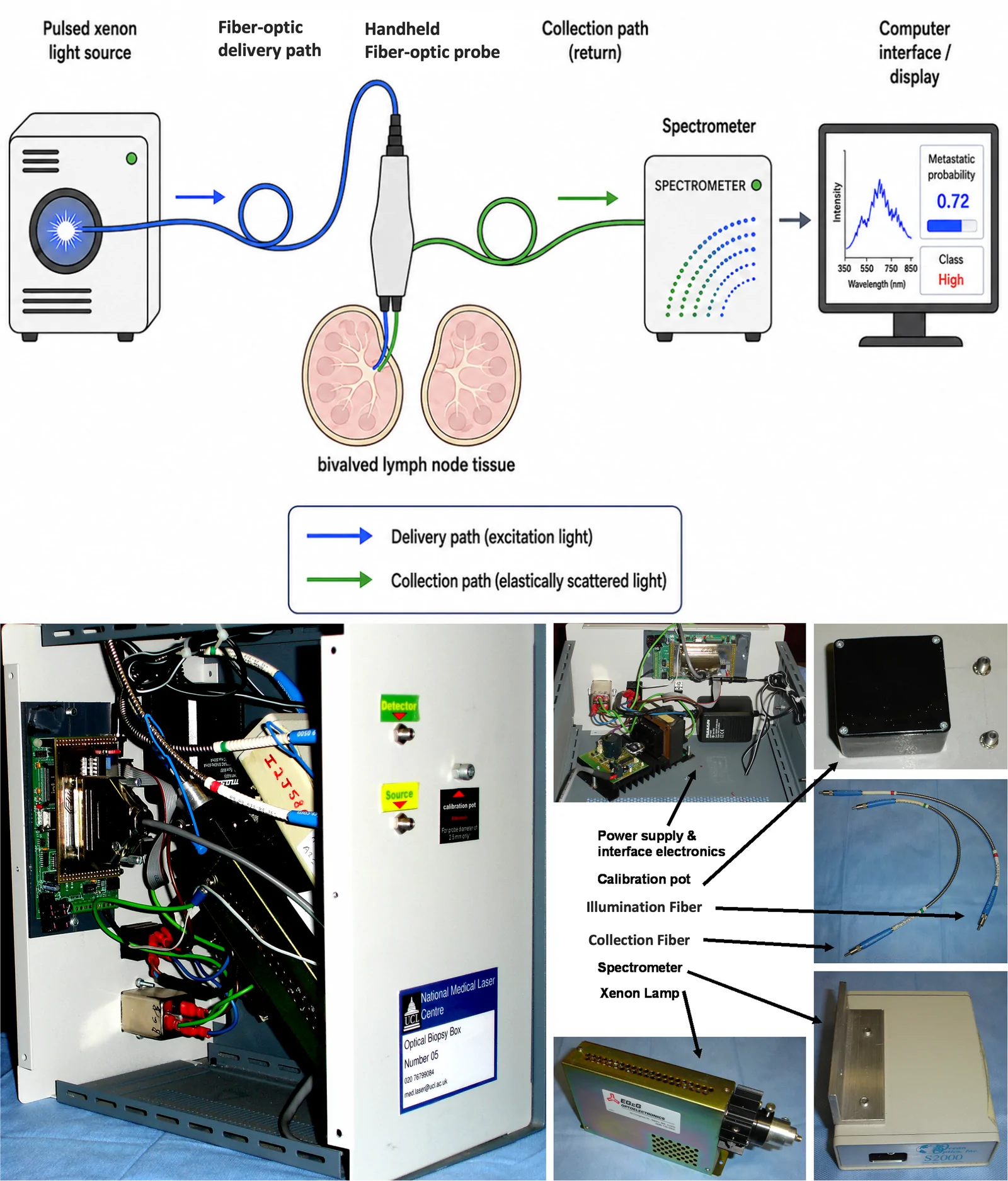
Elastic scattering spectroscopy (ESS) diagnostic system and hardware components. Upper panel: schematic representation of the ESS system used for ex vivo interrogation of bivalved lymph node tissue. Excitation light from a pulsed xenon source is delivered to the tissue through the illumination fiber, shown in blue. The handheld fiber-optic probe is placed in gentle contact with the cut surface of one half of the bivalved lymph node. Elastically scattered light is collected through the adjacent collection fiber and transmitted to the spectrometer, shown in green. Spectral data are then transferred to the computer interface for display and downstream classification. The computer readout is a conceptual illustration of how spectral information and a future model-derived probability could be displayed; it does not represent a prospectively deployed clinical classifier used during specimen acquisition in this study. Lower panel: representative photographs of the ESS hardware, including the optical biopsy box, detector and source ports, calibration port, power supply and interface electronics, illumination and collection fibers, spectrometer, and xenon lamp. Blue arrows indicate the excitation/delivery pathway; green arrows indicate the collected elastically scattered return signal; the gray arrow indicates transfer of spectral information from the spectrometer to the computer display. The system architecture is consistent with the described ESS platform comprising a pulsed xenon arc lamp, fiber-optic probe, spectrometer, and computer-controlled acquisition interface.

The ESS system comprised a pulsed xenon arc lamp light source, a PC-compatible spectrometer based on a modified Ocean Optics platform with linear charge-coupled device array detection, a fiber-optic probe, and a laptop computer for system control and data display. Light was delivered through a 200 micrometer core illumination fiber and scattered light was collected through a neighboring fiber connected to the spectrometer. The probe had a fixed center-to-center separation of approximately 350 micrometers between the delivery and collection fibers. This geometry interrogated a superficial tissue volume of approximately 500 micrometers in length, 300 micrometers in width, and 300 micrometers in depth. The probe was placed in gentle optical contact with the nodal cut surface to minimize reflected light from the surface and to ensure that collected photons had undergone scattering within the tissue.

Before tissue measurements, the system was calibrated using a reflectance standard to normalize the spectral response and reduce variation related to probe transmission, lamp output, and thermal drift. Each acquisition was computer-controlled and included a background measurement followed immediately by pulsed spectral capture with automatic background subtraction. The wavelength range available from the system was broader than the analytical range, but all spectra were subsequently restricted to a common 350‐850 nm window to ensure consistency across measurements. Optical measurement sites were matched to the corresponding lymph node, and the final nodal histopathological diagnosis was used as the class label.

**Figure 2.**
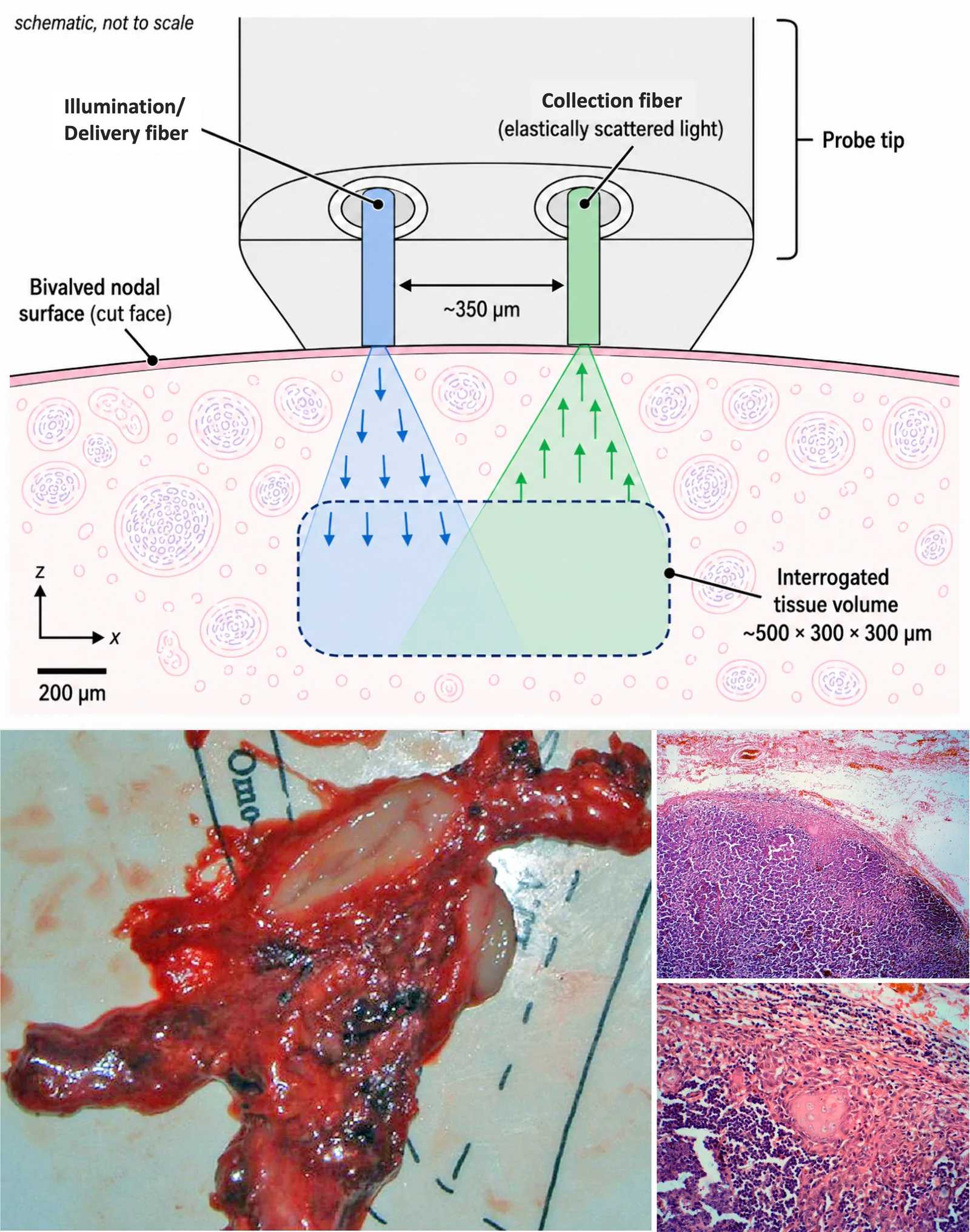
Elastic scattering spectroscopy (ESS) fiber-optic probe geometry, bivalved lymph node sampling, and histopathological correlation. Upper panel: schematic representation of the elastic scattering spectroscopy fiber-optic probe in gentle contact with the exposed cut surface of a bivalved lymph node. The illumination or delivery fiber transmits excitation light into the superficial nodal tissue, shown by the blue cone and downward blue arrows. The adjacent collection fiber captures elastically scattered photons returning from the interrogated tissue volume, shown by the green collection field and upward green arrows. The delivery and collection fibers are separated by approximately 350 µm at the probe tip. With this probe geometry, the sampled optical field interrogates a superficial tissue volume of approximately 500×300×300 µm. The schematic is not drawn to scale and is intended to illustrate probe-tissue geometry rather than exact photon trajectories. Lower left: gross appearance of a surgically excised lymph node specimen after bivalving, exposing the internal nodal surface used for ex vivo optical interrogation. Lower right: representative hematoxylin and eosin-stained sections used for pathological correlation. The upper histological image shows nodal tissue architecture, while the lower histological image demonstrates metastatic keratinizing squamous cell carcinoma within nodal tissue, with malignant squamous nests, keratinization, and associated stromal or inflammatory response. Histopathology remained the reference standard against which the ESS spectral measurements were compared. The histological panels are representative images from the present study material and were not used as quantitative inputs to the ESS classification models.

### Histopathological Reference Standard

After ESS acquisition, each bivalved lymph node was fixed in 10% neutral-buffered formalin for 24‐48 hours, embedded in paraffin, and represented by 1 block from each half. One 4-µm hematoxylin and eosin section was examined from each block. Two deeper levels at 200-µm intervals and pancytokeratin AE1/AE3 immunohistochemistry were used when the initial sections were equivocal or a small metastatic deposit was suspected. The final diagnosis was assigned at lymph-node level.

For this analysis, metastatic deposits were categorized as macrometastases when greater than 2 mm, micrometastases when greater than 0.2 mm and no greater than 2 mm, and isolated tumor cells when no greater than 0.2 mm. Nodes containing macrometastases or micrometastases were classified as pathology positive. No isolated-tumor-cell-only nodes were identified in the cohort. Pathological T and N categories were retained as documented in the original pathology reports. All tumors were retrospectively restaged according to the UICC TNM eighth edition using the recorded depth-of-invasion and extranodal-extension data. Extranodal extension was recorded when metastatic tumor extended beyond the lymph-node capsule.

Pathologists were blinded to the ESS spectra, probability estimates, and model outputs. Routine histopathological assessment was undertaken by consultant pathologists experienced in head and neck pathology using the contemporaneous clinical reporting pathway. Where a requested clinicopathological characteristic was absent from the archived gross or histopathological record, it was recorded as unavailable rather than inferred.

The study cohort comprised 45 patients and 280 ESS-assessed lymph nodes. Of these nodes, 96 were pathology positive and 184 were pathology negative. Three spectra were acquired per node, giving 840 spectra before quality control. Nodes were sampled from both upper and lower neck levels, with 168 nodes from levels I-III and 112 from levels IV-V. The median node size was 14 mm, and acquisition was performed across 9 batches.

Spectra were imported as wavelength-intensity arrays and reviewed using sequential quality-control rules before modeling. Spectra were excluded if they contained zero or failed channels, excessive noise at the spectral edges, clipping or saturation, duplicate entries, or incomplete records. These prespecified quality-control rules were applied before model fitting and without access to the histopathological class label. Of 840 raw spectra, 80 were excluded, leaving 760 retained spectra. At node level, 274 of 280 ESS-assessed nodes retained at least 2 usable spectra and 212 retained all 3 spectra. In each of the remaining 6 pathology-negative nodes, none of the 3 acquired spectra passed the requirement for an analyzable node-level ESS result. These 6 nodes were therefore reported as node-level index-test no-results and were not included in the 274-node modeling and performance denominator. The node-level no-result rate was 2.1% (6/280).

Two complementary preprocessing approaches were used. For feature-based analyses intended to preserve optical amplitude, spectra were lightly smoothed and scaled after restriction to the 350‐850 nm window. For full-spectrum analyses intended to emphasize spectral shape, standard normal variate (SNV) normalization was applied after smoothing. Principal component analysis was used where dimensionality reduction was required, particularly for support vector machine modeling. These preprocessing choices were applied consistently so that amplitude-dominant and shape-dominant analytical strategies could be compared.

A total of 8 candidate optical features were specified before the final comparative modeling analysis on the basis of clinical interpretability and previous ESS work. I360, I450, I630, and I700 were defined as the spectral intensity at the wavelength channel nearest to 360, 450, 630, and 700 nm, respectively. The I630/I360 ratio was calculated from these 2 intensity measurements. Total spectral area was calculated by trapezoidal integration from 350 to 850 nm. Blue trough depth was defined as I450 minus the minimum spectral intensity between 390 and 420 nm. Red shoulder height was defined as the maximum spectral intensity between 650 and 700 nm minus I630. Retained spectra were first summarized by their median within each lymph node so that every node contributed 1 observation per feature. Effect sizes were expressed as rank-biserial correlations. 95% CIs were estimated by resampling patients as clusters while retaining all lymph nodes from each selected patient. Moreover, 2-sided *P* values were obtained by permuting node-level pathology labels within patient while preserving each patient’s number of pathology-positive and pathology-negative nodes. Each permutation-based *P* value was estimated using 10,000 patient-preserving permutations.

### Model Development

Model development compared a baseline I630 rule, in which I630 was defined as the median intensity at the wavelength channel nearest 630 nm across the retained spectra from each lymph node and nodes were classified using a decision threshold selected exclusively from patient-grouped inner-validation training data by maximizing the Youden index, feature-based logistic regression, full-spectrum elastic-net logistic regression, a linear support vector machine, and a hybrid elastic-net model. The amplitude branch comprised the 8 prespecified optical features extracted from Savitzky-Golay–smoothed raw-amplitude spectra, with robust scaling fitted within the inner training folds. The shape branch comprised principal-component scores derived from standard-normal-variate-normalized spectra, with principal component analysis fitted within the inner training folds. Candidate component counts of 10, 20, 30, and 40 were evaluated, with 20 components selected most frequently across the outer fits. The hybrid elastic-net model combined the selected principal-component scores with the 8 scaled optical features. Regularization strength, l1 ratio, and class weighting were selected within the inner folds; C=0.3 and an l1 ratio of 0.5 were the most frequently selected values. Model-family comparisons were exploratory. Each candidate model was tuned independently within the patient-grouped inner validation process, and the hybrid model was carried forward for detailed calibration, error, and robustness analyses because it had the highest pooled patient-grouped out-of-fold area under the receiver operating characteristic curve (AUROC) point estimate. No formal pairwise test of superiority between model families was prespecified.

### Internal Validation and Leakage Control

Primary internal validation used 10 repetitions of nested 5-fold cross-validation implemented using stratified group k-fold splitting, with patient ID as the grouping variable. Within each repetition, pathology class was stratified as closely as possible across folds while all spectra and lymph nodes from an individual patient remained within a single fold. Fold assignment was shuffled before each repetition using random seeds 202601 to 202610, generating 50 outer training-test splits in total. Every spectrum and lymph node from a given patient remained in the same outer fold, so lymph nodes from the same patient could not occur simultaneously in the corresponding outer training and test sets. Within each outer training set, model development and hyperparameter tuning used 4-fold stratified group cross-validation, again grouped by patient and with shuffling enabled. The repetition-specific random seed was retained for the corresponding inner-validation procedure, and no patient represented in an outer test fold was available to any stage of inner model development. Smoothing, scaling, SNV transformation, principal component analysis, component selection, regularization parameters, class weighting, probability calibration where required, and decision threshold selection were fitted or selected using training data only. Outer test patients were used only once the corresponding analytical pipeline had been fixed, solely to generate held-out predictions.

### Model Selection and Decision Threshold

Within each candidate model family, mean inner-fold AUROC was used to select hyperparameters and model configuration. Within each outer training set, the node-level decision threshold was selected from patient-grouped inner-fold out-of-fold predictions using the Youden index. Thus, threshold optimization itself was restricted to training data and did not use the corresponding outer test outcomes. Each node received 1 held-out probability in each of the 10 outer repetitions, and these probabilities were averaged to obtain a single patient-grouped out-of-fold probability for each node. The 50 training-derived thresholds had a median of 0.38 (IQR 0.35‐0.41). For the pooled descriptive confusion matrix and associated threshold-dependent operating characteristics, this median training-derived threshold was applied once to the averaged held-out node probabilities. No pooled outcome or outer-test outcome was used to select or adjust the threshold. Because this single summary threshold was applied after repeated internal cross-validation rather than prospectively fixed before validation, sensitivity, specificity, accuracy, positive predictive value (PPV), and NPV were interpreted as secondary descriptive operating characteristics. AUROC and calibration calculated from the pooled patient-grouped out-of-fold probabilities were the principal performance measures in this exploratory internal-validation analysis. Because these predictions also informed the choice of the hybrid model family, its reported performance estimates may be affected by selection optimism and do not constitute independent validation of a prospectively fixed final model.

### Calibration and Statistical Uncertainty

Calibration was assessed using each node’s averaged patient-grouped out-of-fold probability and was summarized using the Brier score, calibration-in-the-large, calibration slope, and a grouped calibration plot. For the plot, the observed proportion of pathology-positive nodes was compared with the mean predicted probability within each probability group; groups were not constrained to contain equal numbers of nodes. 95% CIs for node-level sensitivity, specificity, accuracy, PPV, NPV, AUROC, and calibration measures were estimated using 2000 patient-clustered bootstrap resamples, with all lymph nodes belonging to each resampled patient retained together. The reported bootstrap intervals were calculated from the completed patient-grouped out-of-fold prediction dataset without refitting the complete model-development procedure within each bootstrap replicate; they therefore quantify uncertainty in the held-out predictions conditional on the completed modeling process. Patient-level CIs for sensitivity, specificity, accuracy, PPV, and NPV were calculated using the Wilson method. The patient-level AUROC CI was estimated by bootstrap resampling of patients.

### Node-Level and Patient-Level Classification

Because the primary diagnostic unit was the lymph node, spectrum-level probabilities were averaged to generate 1 probability for each node. Mean probability across retained spectra was the primary node-level aggregation method. Majority vote, median probability, maximum probability, and classification using a single representative spectrum were evaluated as secondary aggregation strategies. For the single-spectrum analysis, the first quality-control-passing acquisition from each lymph node according to the recorded acquisition order was selected, without reference to histopathological status or model output.

A secondary patient-level analysis was undertaken using the cross-validation–derived summary node-level threshold. A patient was reference-standard positive when at least 1 ESS-assessed lymph node was metastatic on histopathology and model positive when at least 1 assessed node exceeded the summary threshold. The maximum averaged out-of-fold node probability within each patient was used for patient-level receiver operating characteristic curve analysis. The patient-level analysis therefore represented only the ESS-assessed node set and was not interpreted as complete pathological staging of the neck.

Performance was summarized using sensitivity, specificity, accuracy, PPV, NPV, and AUROC. Robustness analyses included leave-one-patient-out validation, leave-one-batch-out validation, exclusion of the lowest-quality 10% of retained spectra ranked by signal-to-noise ratio, use of the first 2 quality-control–passing spectra from each node according to recorded acquisition order, restriction to nodes retaining all 3 spectra, area-normalized spectra, and shape-focused preprocessing.

### Analysis Plan and Reproducibility

The 3-acquisition sampling protocol and 8 candidate optical features were specified before the final modeling analysis. The comparison of machine learning algorithms, model-family selection, and associated hyperparameter grids was exploratory and was not prospectively registered. Accordingly, comparisons between candidate model families were interpreted descriptively, and no claim of formal superiority was made. Analyses were conducted using Python (version 3.12.8; Python Software Foundation), *NumPy* 2.2.1, *pandas* 2.2.3, *SciPy* 1.15.0, and *scikit-learn* 1.6.0. Random seeds 202601 to 202610 were used for the 10 outer repetitions. No formal sample-size calculation was undertaken; the analysis used the complete available cohort meeting the eligibility requirements and having linked ESS and histopathological data.

Reporting followed TRIPOD (Transparent Reporting of a Multivariable Prediction Model for Individual Prognosis or Diagnosis)+AI, STARD (Standards for Reporting of Diagnostic Accuracy Studies)-AI, and STARD 2015 [17,18,21]. Completed reporting checklists are provided in Checklists 1-3 Table S1 and Figure S1 in Multimedia Appendix 1 provide the analytical workflow, software versions, preprocessing sequence, candidate component counts, hyperparameter grid, threshold-selection method, and leakage-control procedures.

## Results

### Overview

In total, 52 patients were assessed for eligibility. Of the total, 7 were excluded before enrollment; 3 because consent was not obtained, 2 because no suitable fresh lymph nodes were available before fixation, and 2 because the ESS system was unavailable at the time of surgery. The final cohort comprised 45 patients, with a median age of 61 (IQR 53‐69) years; 29 were male and 16 were female. Moreover, 25 patients had at least 1 pathology-positive ESS-assessed node, and 20 had no positive assessed nodes. Primary tumor site, pathological T category, clinical and pathological N category, depth of invasion, histological grade, extranodal extension, and positive-node burden are summarized in Table 1.

**Table 1.** Participant and primary tumor characteristics. Percentages may not total 100 because of rounding.

Characteristic	Overall cohort
Patients, n	45
Age (y), median (IQR)	61 (53‐69)
Age range (y)	34‐82
Sex, n (%)	
Male	29 (64.4)
Female	16 (35.6)
Primary tumor site, n (%)	
Oral tongue	17 (37.8)
Floor of mouth	9 (20)
Mandibular alveolus	7 (15.6)
Buccal mucosa	5 (11.1)
Retromolar trigone	4 (8.9)
Maxillary alveolus or hard palate	3 (6.7)
Pathological T category, n (%)	
pT1	8 (17.8)
pT2	17 (37.8)
pT3	8 (17.8)
pT4a	12 (26.7)
Clinical N category, n (%)	
cN0	23 (51.1)
cN1	9 (20)
cN2	13 (28.9)
Pathological N category, n (%)	
pN0	20 (44.4)
pN1	8 (17.8)
pN2	17 (37.8)
Depth of invasion (mm), median (IQR)	8 (5-13)
Histological grade, n (%)	
Well differentiated	8 (17.8)
Moderately differentiated	29 (64.4)
Poorly differentiated	8 (17.8)
Patients with ≥1 pathology-positive ESS-assessed^a^ node, n (%)	25 (55.6)
Extranodal extension among node-positive patients, n/N (%)	9/25 (36)
Positive nodes per node-positive patient, median (IQR); range	3 (2-5); 1‐9

a ESS: elastic scattering spectroscopy.

The patients contributed 280 ESS-assessed cervical lymph nodes, of which 96 were pathology positive and 184 were pathology negative. The median node size was 14 (IQR 9‐21) mm. Levels I-III accounted for 168 nodes and levels IV-V for 112 nodes. The median number of ESS-assessed nodes per patient was 6 (IQR 4‐8). The study timeline, participant flow, nodal sampling, and acquisition characteristics are summarized in Table 2.

**Table 2.** Study timeline, participant flow, nodal sampling, and acquisition characteristics. The REC^a^ dates refer to the optical-imaging study. The study scope, provisions for annual local organizational governance checks, and responsibilities of the Chief Investigator are described in the Ethical Considerations section.

Variable	Value
Study timeline	
Optical-imaging study: REC application submitted	April 26, 2006
Optical-imaging study: favorable ethical opinion confirmed, REC 06/Q0505/39	July 31, 2006
Surgery, tissue collection, and ESS^b^ acquisition	September 2016-March 2021
Data curation	January-December 2025
Exploratory model development and internal validation	January-May 2026
Participant flow	
Patients assessed for eligibility, n	52
Patients excluded before enrollment, n (%)	7 (13.5)
Written consent not obtained, n	3
No suitable fresh lymph nodes available before fixation, n	2
ESS system unavailable at the time of surgery, n	2
Patients included, n	45
Nodal sampling	
Lymph nodes assessed by ESS, n	280
Pathology-positive nodes, n (%)	96 (34.3)
Pathology-negative nodes, n (%)	184 (65.7)
Nodes from neck levels I-III, n (%)	168 (60)
Nodes from neck levels IV-V, n (%)	112 (40)
Node size (mm), median (IQR)	14 (9‐21)
ESS-assessed nodes per patient, median (IQR)	6 (4-8)
Acquisition characteristics	
ESS spectra acquired, n	840
Spectra acquired per node, n	3
Acquisition batches, n	9
Operator A	31 patients; 193 nodes
Operator B	14 patients; 87 nodes
Time from excision to first ESS acquisition (min), median (IQR)	18 (13‐26)
Time from excision to first ESS acquisition (min), range	7‐48

a REC:

b ESS: elastic scattering spectroscopy.

### Acquisition and Quality Control

A total of 3 spectra were acquired from each ESS-assessed lymph node, giving 840 acquisitions. Quality control excluded 80 spectra—42 because of zero or failed channels, 19 because of excessive spectral-edge noise, 11 because of clipping or saturation, and 8 because of duplicate or incomplete records. The final spectrum set comprised 760 retained spectra, equivalent to 90.5% of acquisitions.

At node level, 274 lymph nodes retained at least 2 usable spectra and entered the analytical cohort. These comprised 96 pathology-positive and 178 pathology-negative nodes. In each of 6 additional pathology-negative nodes, none of the 3 acquired spectra met the requirement for an analyzable node-level ESS result. These 6 nodes were reported as node-level index-test no-results and were not included in the 274-node modeling or performance denominator. Moreover, 212 nodes retained all 3 spectra. Participant, specimen, acquisition, and analysis flow is illustrated in Figure 3, and quality-control outcomes are reported in Table 3.

**Figure 3.**
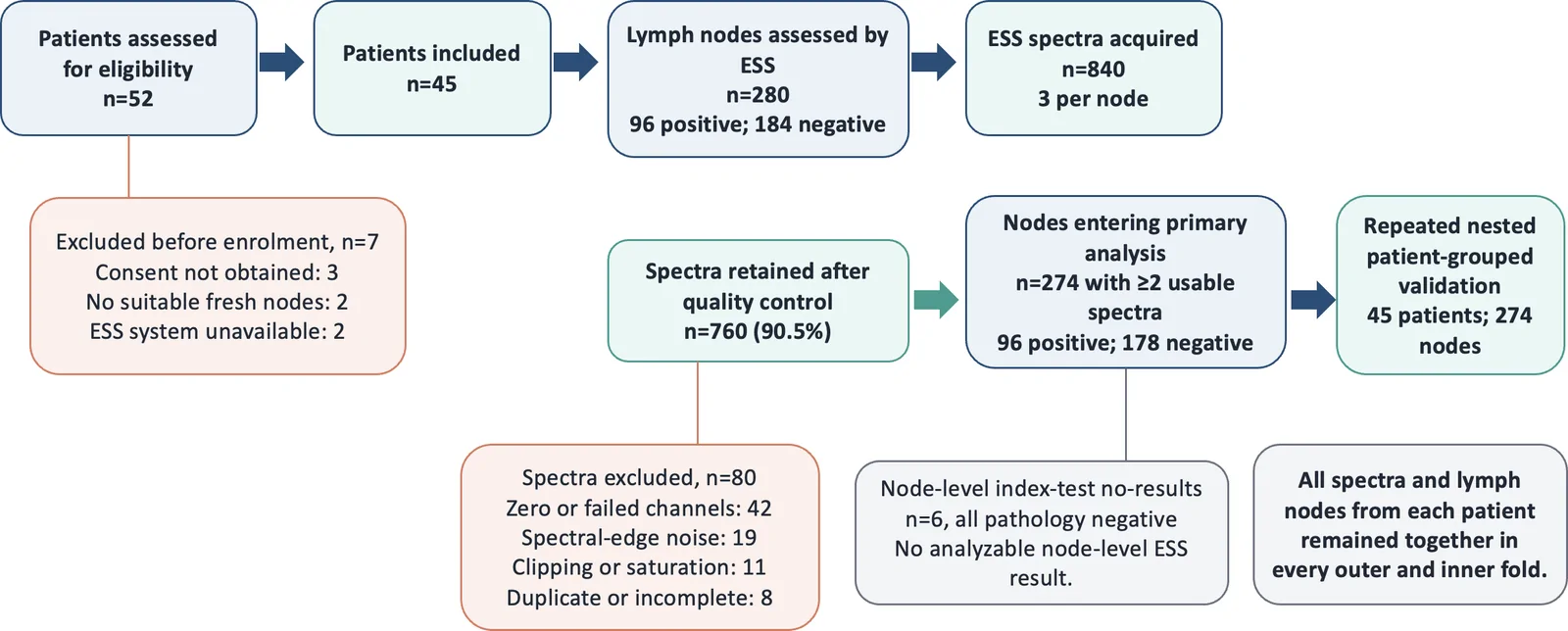
Participant, specimen, acquisition, and analysis flow. Of 52 patients assessed for eligibility, 7 were excluded before enrollment: 3 because consent was not obtained, 2 because no suitable fresh lymph nodes were available before fixation, and 2 because the elastic scattering spectroscopy (ESS) system was unavailable at the time of surgery. In total, 45 patients were included and contributed 280 ESS-assessed lymph nodes, of which 96 were pathology positive and 184 were pathology negative. Moreover, 3 spectra were acquired from each node, giving 840 acquisitions. Quality control excluded 80 spectra, leaving 760 retained spectra. At node level, 274 nodes retained at least 2 usable spectra and comprised the analytical cohort; these included 96 pathology-positive and 178 pathology-negative nodes. Furthermore, 6 pathology-negative nodes produced no analyzable node-level ESS result and were reported as index-test no-results. All spectra and lymph nodes from each patient remained together during patient-grouped internal validation.

The 6 node-level index-test no-results were pathology negative and did not contribute to the 274-node modeling or performance denominator. Percentages for spectrum-level quality-control exclusions use 840 acquired spectra as the denominator; node percentages use 280 ESS-assessed nodes unless otherwise stated. Percentages shown for pathology-positive and pathology-negative analyzable nodes use 274 nodes as the denominator.

**Table 3.** Spectral quality control and node-level index-test no-results. The 6 node-level index-test no-results were pathology negative and did not contribute to the 274-node modeling or performance denominator. Percentages for spectrum-level quality-control exclusions use 840 acquired spectra as the denominator; node percentages use 280 elastic scattering spectroscopy–assessed nodes unless otherwise stated. Percentages shown for pathology-positive and pathology-negative analyzable nodes use 274 nodes as the denominator.

Quality-control step	Values, n (%)
Raw spectra acquired	840 (100)
Spectra with zero or failed channels	42 (5)
Spectra excluded for excessive spectral-edge noise	19 (2.3)
Spectra excluded for clipping or saturation	11 (1.3)
Spectra excluded as duplicate or incomplete records	8 (1)
Total excluded spectra	80 (9.5)
Spectra retained	760 (90.5)
Nodes retaining ≥2 usable spectra	274 (97.9)
Pathology positive	96 (35 of analyzed nodes)
Pathology negative	178 (65 of analyzed nodes)
Nodes retaining all 3 spectra	212 (75.7)
Node-level index-test no-results	6 (2.1)

### Spectral Feature Differences

Pathology-positive lymph nodes showed higher intensities at 360, 450, and 630 nm and greater total spectral area. In contrast, pathology-negative nodes showed higher intensity at 700 nm, a higher I630/I360 ratio, a deeper local blue trough, and a larger red shoulder. The differences were therefore bidirectional rather than representing a uniform increase in spectral amplitude and supported the inclusion of both amplitude-related and shape-related information. The node-level median ESS profiles are illustrated in Figure 4, and numerical medians and IQRs for the prespecified node-level spectral features are reported in Table 4.

**Figure 4.**
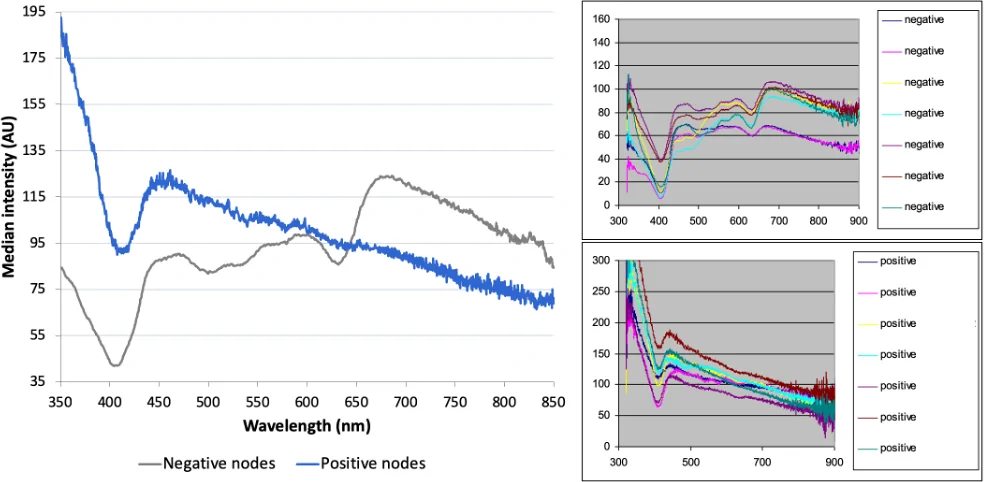
Median and representative elastic scattering spectroscopy profiles of pathology-negative and pathology-positive lymph nodes. The left panel shows node-level median elastic scattering spectroscopy (ESS) profiles across the 350-850 nm analytical range. Numerical medians and IQRs for the prespecified node-level spectral features are reported in Table 4. The pathology-positive profile showed higher short-wavelength intensity and a smoother, more gradually declining pattern, whereas the pathology-negative profile showed a deeper local blue trough and a later rise in the red and near-red region. The upper-right and lower-right panels show representative raw spectra generated by the ESS acquisition software for pathology-negative and pathology-positive nodes, respectively, illustrating within-class acquisition variability. The raw-spectrum panels retain the native acquisition-software wavelength and intensity scales and should not be used for direct between-class amplitude comparison. AU: arbitrary unit.

**Table 4.** Node-level spectral feature differences by pathological status. Spectra were first aggregated within each lymph node. Positive rank-biserial correlations indicate higher values in pathology-positive nodes, whereas negative correlations indicate higher values in pathology-negative nodes. CIs were estimated by resampling patients while retaining all lymph nodes from each selected patient. Blue trough depth was calculated as I450 minus the minimum intensity from 390 to 420 nm. Red shoulder height was calculated as the maximum intensity from 650 to 700 nm minus I630. The reported *P* values are exploratory, unadjusted for multiplicity, and should be interpreted together with the corresponding effect estimates and CIs rather than as confirmatory hypothesis tests.

Feature	Pathology-negative nodes, median (IQR)	Pathology-positive nodes, median (IQR)	Rank-biserial correlation (95% CI)	*P* value
I360, AU^a^	80 (69‐92)	168 (149‐186)	0.88 (0.82 to 0.92)	<.001
I450, AU	85 (76‐95)	121 (109‐134)	0.69 (0.59 to 0.77)	<.001
I630, AU	88 (84‐92)	96 (93‐103)	0.80 (0.72 to 0.86)	<.001
I700, AU	120 (107‐132)	88 (81‐96)	–0.78 (–0.84 to –0.69)	<.001
I630/I360 ratio	1.10 (0.99‐1.22)	0.58 (0.53‐0.66)	–0.86 (–0.90 to –0.79)	<.001
Area, 350‐850 nm, AU·nm	45,800 (41,600-50,700)	50,600 (46,900-55,400)	0.44 (0.30 to 0.56)	<.001
Blue trough depth, approximately 400 nm, AU	43.1 (35.8‐50.5)	31.4 (25.6‐38.2)	–0.24 (–0.38 to –0.09)	.002
Red shoulder height, 650‐700 nm, AU	31.6 (24.8‐38.9)	6.7 (3.9‐10.2)	–0.82 (–0.87 to –0.75)	<.001

a AU: arbitrary unit.

Pathology-positive nodes had higher I360, I450, and I630 intensities and greater total spectral area, whereas pathology-negative nodes had higher I700 intensity, a higher I630/I360 ratio, a deeper blue trough, and a larger red shoulder. The largest effects were observed for I360, with a rank-biserial correlation of 0.88 (95% CI 0.82‐0.92); the I630/I360 ratio, with a correlation of –0.86 (95% CI –0.90 to –0.79); red shoulder height, with a correlation of –0.82 (95% CI –0.87 to –0.75); I630, with a correlation of 0.80 (95% CI 0.72‐0.86); and I700, with a correlation of –0.78 (95% CI –0.84 to –0.69). Moreover, 7 features had *P*<.001, while blue trough depth remained significant at *P*=.002. Full medians, IQRs, effect sizes, CIs, and *P* values are reported in Table 4 and Figure 5.

**Figure 5.**
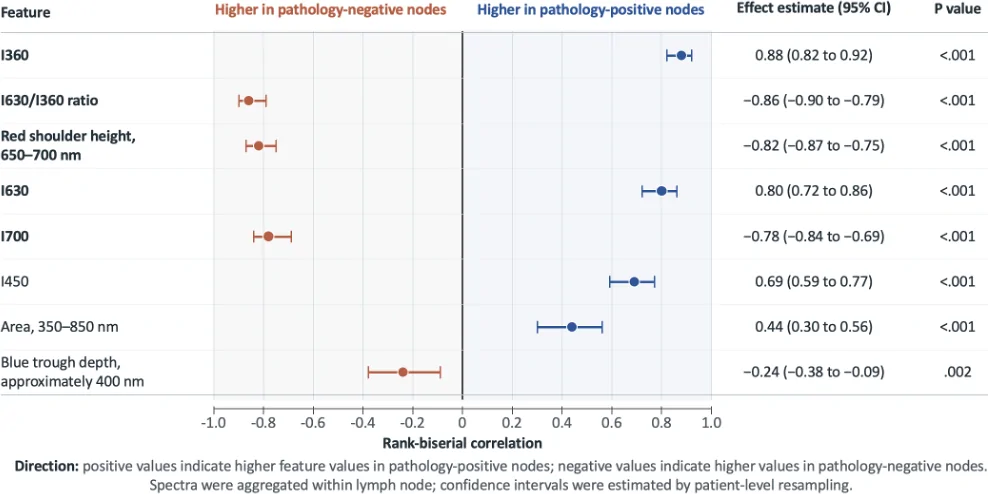
Effect sizes for prespecified node-level spectral features. Points show rank-biserial correlations, and horizontal lines show patient-clustered 95% CIs. Positive correlations indicate higher feature values in pathology-positive nodes, whereas negative correlations indicate higher values in pathology-negative nodes. Features are ordered by the absolute magnitude of the effect estimate. Spectra were first aggregated within each lymph node, and CIs were estimated by resampling patients while retaining all lymph nodes from each selected patient. The corresponding *P* values were derived from patient-preserving permutation analyses and are exploratory and unadjusted for multiplicity.

### Patient-Grouped Internal Validation

Repeated nested patient-grouped cross-validation yielded an internally validated, exploratory AUROC estimate of 0.966 (95% CI 0.929‐0.988) for the selected hybrid model. Because the hybrid model family was selected using the same pooled out-of-fold predictions, this result is not an independent validation estimate for a prospectively fixed final model. At the cross-validation–derived summary threshold, the pooled descriptive confusion matrix comprised 90 true-positive, 6 false-negative, 170 true-negative, and 8 false-positive lymph nodes. The corresponding secondary descriptive operating characteristics were as follows: sensitivity 93.8% (95% CI 86.4%‐97.6%), specificity 95.5% (95% CI 90.1%‐98.3%), accuracy 94.9% (95% CI 90.4%‐97.3%), PPV 91.8% (95% CI 82.9%‐96.3%), and NPV 96.6% (95% CI 92.4%‐98.8%). The 50 training-derived decision thresholds had a median of 0.38 (IQR 0.35‐0.41); no pooled outcome or outer-test outcome was used to select or modify this threshold. The Brier score was 0.063, calibration-in-the-large was –0.03, and the calibration slope was 0.94. The calibration plot showed close agreement between observed and predicted probabilities across most of the probability range, with modest overprediction at the highest probabilities. Classification and calibration results are presented in Table 5 and Figure 6.

**Table 5.** Selected hybrid model discrimination, descriptive operating characteristics, calibration, aggregation, and robustness. Threshold-dependent measures are presented as secondary descriptive operating characteristics. For the primary patient-grouped node-level analysis, the cross-validation–derived summary threshold was applied to each node’s averaged held-out probability to obtain the pooled confusion matrix. For the patient-grouped spectrum-level analysis and the analysis restricted to nodes retaining all 3 spectra, sensitivity, specificity, and accuracy were calculated separately within each of the 10 outer repetitions and then averaged; these secondary percentages therefore represent means of repetition-specific metrics rather than proportions derived from a single pooled confusion matrix. AUROC^a^ estimates were derived from held-out probabilities within the reported internal-validation analyses. The primary patient-grouped AUROC of 0.966 is an internally validated, exploratory estimate for a model family selected using the same pooled out-of-fold predictions and does not represent independent validation of a prospectively fixed final model. Patient-level PPV^b^ was 92.3% (95% CI 75.9%-97.9%) and NPV^c^ was 94.7% (95% CI 75.4%-99.1%). Patient-level CIs for sensitivity, specificity, accuracy, PPV, and NPV were calculated using the Wilson method; the patient-level AUROC CI was estimated by bootstrap resampling of patients.

Domain or analysis	Analytical set	Sensitivity,%	Specificity,%	Accuracy,%	AUROC	Additional result
Validation level						
Primary patient-grouped node-level analysis	274 nodes	93.8	95.5	94.9	0.966	90 TP^d^; 6 FN^e^; 170 TN^f^; 8 FP^g^
Secondary node-grouped node-level analysis	274 nodes	96.9	96.6	96.7	0.989	93 TP; 3 FN; 172 TN; 6 FP
Patient-grouped spectrum-level analysis	760 spectra	89.6	92.0	91.2	0.944	Secondary analysis
Secondary patient-level analysis	45 patients	96.0	90.0	93.3	0.956	24 TP; 1 FN; 18 TN; 2 FP
Calibration of the primary hybrid model						
Brier score	274 nodes	—^h^	—	—	—	0.063 (95% CI 0.044‐0.086)
Calibration-in-the-large	274 nodes	—	—	—	—	−0.03 (95% CI −0.21 to 0.15)
Calibration slope	274 nodes	—	—	—	—	0.94 (95% CI 0.74‐1.17)
Training-derived threshold distribution	Outer splits	—	—	—	—	Median 0.38 (IQR 0.35‐0.41) across 50 outer training sets
Node-level aggregation						
Single prespecified spectrum	274 nodes	88.5	89.9	89.4	0.930	—
Majority vote	274 nodes	92.7	92.7	92.7	0.953	—
Mean probability	274 nodes	93.8	95.5	94.9	0.966	Primary method
Median probability	274 nodes	92.7	94.9	94.2	0.963	—
Maximum probability	274 nodes	97.9	87.6	91.2	0.959	Higher false-positive rate
Robustness analyses						
Leave-one-patient-out validation	274 nodes	92.7	94.9	94.2	0.961	—
Leave-one-batch-out validation	274 nodes	91.7	93.8	93.1	0.952	—
Excluding the lowest-quality 10% of spectra	266 nodes	94.7	95.9	95.5	0.969	—
Using 2 spectra per node	274 nodes	91.7	93.8	93.1	0.951	—
Nodes retaining all 3 spectra	212 nodes	94.7	95.6	95.3	0.964	—
Area-normalized spectra	274 nodes	85.4	87.1	86.5	0.895	—
SNV^i^ plus first derivative	274 nodes	87.5	89.3	88.7	0.912	—

a AUROC: area under the receiver operating characteristic curve.

b PPV: positive predictive value.

c NPV: negative predictive value.

d TP: true positive.

e FN: false negative.

f TN: true negative.

g FP: false positive.

h Not applicable.

i SNV: standard normal variate.

Among the candidate model families, the hybrid model had the highest pooled patient-grouped out-of-fold AUROC point estimate. Comparative performance is reported in Table 6 and Figure 7. Because model-family comparisons were exploratory and no formal pairwise superiority test was prespecified, these differences should not be interpreted as establishing superiority of one model family over another.

In the secondary node-grouped analysis, the hybrid model produced 93 true-positive, 3 false-negative, 172 true-negative, and 6 false-positive nodes. These counts corresponded to sensitivity of 96.9%, specificity of 96.6%, accuracy of 96.7%, PPV of 93.9%, NPV of 98.3%, and an AUROC of 0.989. The higher point estimates obtained when grouping was restricted to lymph nodes illustrate the potential for optimistic performance estimates when patient-level clustering is not preserved.

**Figure 6.**
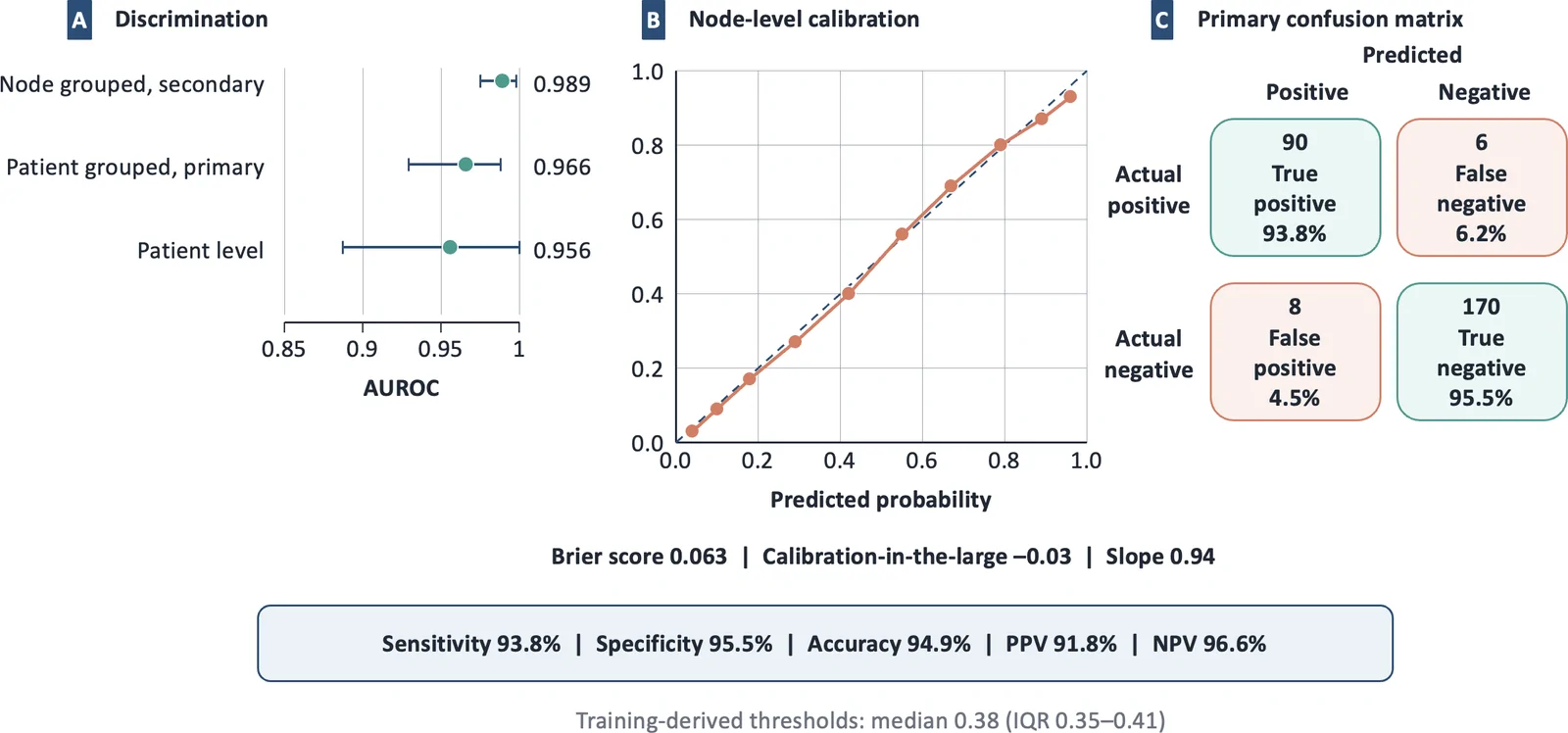
Exploratory discrimination, calibration, and descriptive classification performance of the selected hybrid model under internal validation. Panel A shows area under the receiver operating characteristic curve (AUROC) estimates for the primary patient-grouped node-level analysis, the secondary node-grouped analysis, and the patient-level analysis. The primary patient-grouped AUROC of 0.966 is an internally validated, exploratory estimate for the selected model and does not represent independent validation of a prospectively fixed final model. Points show AUROC estimates, and horizontal bars show the corresponding 95% CIs for the primary patient-grouped node-level analysis, the secondary node-grouped analysis, and the patient-level analysis. Panel B compares observed pathology-positive proportions with mean predicted probabilities calculated from averaged patient-grouped out-of-fold node predictions. Each point represents a probability group, and the groups were not constrained to contain equal numbers of nodes. The diagonal line represents perfect calibration. The Brier score was 0.063, calibration-in-the-large was –0.03, and the calibration slope was 0.94. Panel C shows the descriptive confusion matrix obtained by applying the cross-validation-derived summary threshold to averaged patient-grouped held-out probabilities: 90 true-positive, 6 false-negative, 170 true-negative, and 8 false-positive nodes. The 50 training-derived thresholds had a median of 0.38 (IQR 0.35-0.41). No pooled outcome or outer-test outcome was used to select or adjust the summary threshold. NPV: negative predictive value; PPV: positive predictive value.

**Table 6.** Patient-grouped internal validation of candidate node-level models. AUROC^a^ estimates were derived from pooled patient-grouped out-of-fold predictions and represent exploratory internal-validation performance. The hybrid model family was selected on the basis of these same predictions, its AUROC of 0.966 should therefore not be interpreted as independent validation of a prospectively fixed final model. For each candidate model family, sensitivity, specificity, accuracy, PPV^b^, and NPV^c^ were calculated using that model family’s own summary threshold derived from patient-grouped inner-validation predictions and are presented as secondary descriptive operating characteristics. 95% CIs were estimated using 2000 patient-clustered bootstrap resamples. Model-family comparisons were exploratory and no formal pairwise superiority testing was prespecified.

Model	Sensitivity,% (95% CI)	Specificity,% (95% CI)	Accuracy,% (95% CI)	PPV,% (95% CI)	NPV,% (95% CI)	AUROC (95% CI)
I630 rule	84.4 (75.0‐91.0)	87.1 (79.8‐92.4)	86.1 (80.4‐90.2)	77.9 (65.8‐86.4)	91.2 (84.9‐95.0)	0.902 (0.852‐0.938)
Feature logistic regression	88.5 (79.5‐94.6)	90.4 (83.2‐94.8)	89.8 (84.5‐93.6)	83.3 (73.0‐90.2)	93.6 (88.0‐97.0)	0.936 (0.892‐0.964)
Elastic-net full-spectrum model	92.7 (84.4‐97.1)	93.3 (87.0‐97.2)	93.1 (88.3‐96.4)	88.1 (78.5‐94.8)	96.0 (91.2‐98.8)	0.960 (0.925‐0.980)
Linear support vector machine	91.7 (83.2‐96.5)	92.1 (85.7‐96.5)	92.0 (86.9‐95.8)	86.3 (76.2‐93.5)	95.3 (90.4‐98.4)	0.954 (0.918‐0.976)
Hybrid elastic-net model	93.8 (86.4‐97.6)	95.5 (90.1‐98.3)	94.9 (90.4‐97.3)	91.8 (82.9‐96.3)	96.6 (92.4‐98.8)	0.966 (0.929‐0.988)

a AUROC: area under the receiver operating characteristic curve.

b PPV: positive predictive value.

c NPV: negative predictive value.

**Figure 7.**
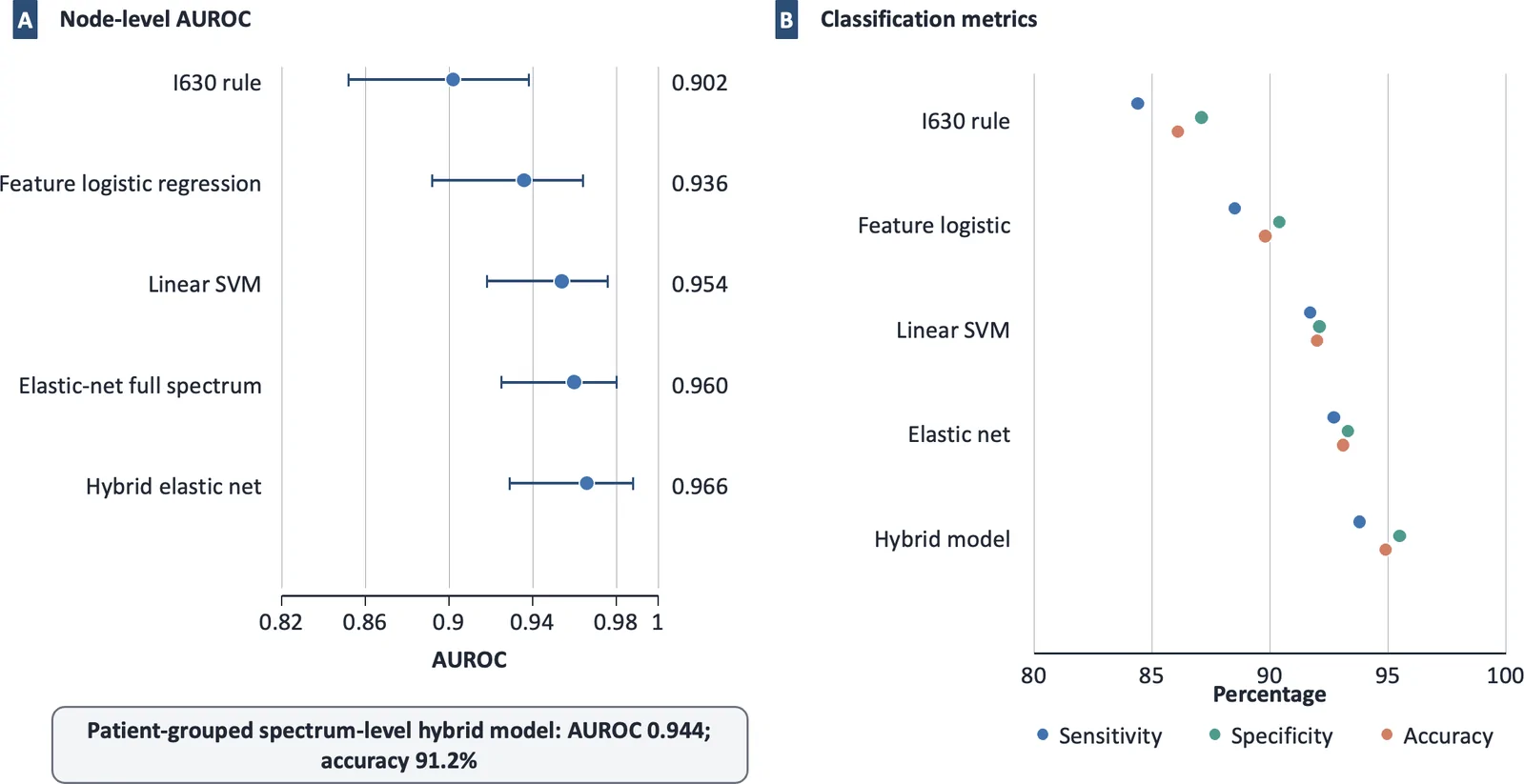
Exploratory comparison of candidate elastic scattering spectroscopy (ESS) models under patient-grouped internal validation. Panel A shows node-level area under the receiver operating characteristic curve (AUROC) and 95% CIs for the I630 rule, feature logistic regression, linear support vector machine, elastic-net full-spectrum model, and hybrid elastic-net model. Panel B shows the corresponding descriptive sensitivity, specificity, and accuracy estimates at each model family’s own cross-validation-derived summary threshold. The hybrid elastic-net model family was selected because it had the highest pooled patient-grouped out-of-fold AUROC point estimate among the candidates. Its AUROC of 0.966 is an internally validated, exploratory estimate based on the same predictions used for model-family selection, rather than independent validation of a prospectively fixed final model. No formal pairwise superiority test was prespecified. The patient-grouped spectrum-level hybrid analysis had an AUROC of 0.944 and a mean accuracy of 91.2% across the 10 outer repetitions. SVM: support vector machine.

### Patient-Level Performance

At patient level, 25 patients were reference-standard positive and 20 were negative within the ESS-assessed node set. Using the cross-validation–derived summary node-level threshold, 24 of 25 positive patients and 18 of 20 negative patients were classified correctly, giving 1 false-negative and 2 false-positive patient classifications. Patient-level sensitivity was 96% (95% CI 80.5%‐99.3%), specificity was 90% (95% CI 69.9%‐97.2%), accuracy was 93.3% (95% CI 82.1%‐97.7%), PPV was 92.3% (95% CI 75.9%‐97.9%), and NPV was 94.7% (95% CI 75.4%‐99.1%). The patient-level AUROC, based on the maximum node probability within each patient, was 0.956 (95% CI 0.887‐1.000).

Patient-grouped spectrum-level analysis produced the same model ranking but lower performance than node-level analysis. The spectrum-level hybrid model had an AUROC of 0.944; mean sensitivity, specificity, and accuracy across the 10 outer repetitions were 89.6%, 92%, and 91.2%, respectively. This reduction supported the use of repeated acquisition and node-level aggregation rather than classification of individual spectra as independent diagnostic observations. Spectrum-level results are reported in Table 5 and Figure 7.

### Aggregation and Robustness Analyses

Mean probability across retained spectra was the prespecified primary node-level aggregation method and provided a favorable balance of sensitivity and specificity in this analysis. Under patient-grouped validation, a single representative spectrum achieved accuracy of 89.4% and an AUROC of 0.930, while majority vote achieved accuracy of 92.7% and an AUROC of 0.953. Median probability performed similarly to mean probability. Maximum probability increased sensitivity to 97.9% but reduced specificity to 87.6%, resulting in more false-positive classifications.

The primary result remained stable in leave-one-patient-out validation, which produced an AUROC of 0.961, and leave-one-batch-out validation, which produced an AUROC of 0.952. Excluding the lowest-quality 10% of spectra produced an AUROC of 0.969. Restriction to 2 spectra per node reduced the AUROC to 0.951, whereas restriction to nodes retaining all 3 spectra gave an AUROC of 0.964. Area-normalized and shape-focused analyses produced lower AUROCs of 0.895 and 0.912, respectively, indicating that amplitude information contributed materially to discrimination. Full results are reported in Table 5.

### Error Analysis

Using the cross-validation–derived summary threshold, the primary patient-grouped analysis classified 260 of 274 analyzable lymph nodes correctly, including 90 true-positive and 170 true-negative nodes. Moreover, 14 analyzable nodes were misclassified; 6 were false negative and 8 were false positive. The additional 6 pathology-negative ESS-assessed nodes that produced no analyzable node-level ESS result were reported separately as index-test no-results and were not included among the 14 model misclassifications.

Of the 6 false-negative nodes, 4 contained micrometastases measuring 0.3‐1.6 mm, while 2 contained small peripheral macrometastases measuring 2.3 mm and 2.8 mm. The median false-negative node size was 8.5 mm, and none showed extranodal extension. The 8 false-positive nodes were clustered within 2 pathology-negative patients and showed marked reactive or inflammatory changes, including follicular hyperplasia, sinus histiocytosis, vascular congestion, surface hemorrhage, or suppurative inflammation. Summary findings are reported in Table 7, and detailed case-level findings are reported in Table S2 in Multimedia Appendix 1.

**Table 7.** Error analysis of the selected patient-grouped hybrid model. Misclassification status was determined using the cross-validation–derived summary node-level threshold of 0.38. The 6 pathology-negative node-level index-test no-results were reported separately and are not included in this table. Detailed case-level findings are reported in Table S2 in Multimedia Appendix 1.

Error category	n	Node size (mm), median (range)	Deposit size (mm), median (range)	Metastatic class	Neck level	Histopathological context	Out-of-fold probability, median (range)
False negative	6	8.5 (5-13)	1.2 (0.3‐2.8)	4 micrometastases; 2 small peripheral macrometastases	4 at levels I-II; 2 at levels III-IV	Reactive change in 2; focal necrosis in 1; keratinization in 1; extranodal extension in 0	0.113 (0.028‐0.239)
False positive	8	16.5 (9-22)	—^a^	All pathology negative	5 at levels I-II; 3 at levels III-IV	All arose in 2 patients with marked reactive or inflammatory change; focal necrosis in 1; keratinization in 0	0.915 (0.420‐0.995)

a Not applicable.

### Technical Repeatability and Variability

Within-node agreement across the 3 spatially separated acquisitions was highest for I630 intensity, with an intraclass correlation coefficient (ICC) of 0.91 (95% CI 0.88‐0.93) and a median coefficient of variation of 7.4%. The corresponding ICCs were 0.89 for total spectral area, 0.84 for red shoulder height, 0.78 for blue trough depth, and 0.87 for spectrum-level hybrid probability. Intraclass correlation coefficients were calculated using a 1-way random-effects, absolute-agreement, single-measure model (ICC 1,1), with 95% CIs estimated from the corresponding F distribution.

Patient-grouped AUROC was 0.968 for acquisitions undertaken by operator A and 0.961 for acquisitions undertaken by operator B. The difference was 0.007 (95% CI –0.031 to 0.044). Acquisitions completed within 30 minutes of excision produced an AUROC of 0.967, compared with 0.958 for acquisitions completed after 30 minutes. Leave-one-batch-out validation produced an AUROC of 0.952. Because each lymph node was acquired by a single operator, the operator analysis compared independent operator-specific case series rather than paired measurements of the same nodes and therefore did not estimate interoperator reproducibility directly. Operator, batch, and timing analyses were interpreted as sensitivity analyses because differences in patient and nodal case mix could not be fully separated from technical effects. Results are reported in Table S3 and Figure S2 in Multimedia Appendix 1.

## Discussion

### Principal Findings

This single-center model-development and internal-validation study found that ESS could discriminate metastatic from nonmetastatic cervical lymph nodes ex vivo. The selected hybrid model had an internally validated, exploratory AUROC estimate of 0.966 under patient-grouped cross-validation. This estimate may be affected by selection optimism because the same pooled out-of-fold predictions informed both model-family selection and performance reporting, and it should not be interpreted as independent validation of a prospectively fixed final model. At the cross-validation–derived summary threshold, secondary descriptive operating characteristics were sensitivity 93.8%, specificity 95.5%, and accuracy 94.9%. By comparison, validation grouped only at lymph-node level produced an AUROC of 0.989 and accuracy of 96.7%. The lower patient-grouped estimates illustrate the importance of preserving patient-level clustering when multiple lymph nodes originate from the same individual. The clinically relevant model output was a lymph-node probability derived from repeated spectra rather than classification of individual spectral acquisitions as independent observations.

Pathology-positive nodes showed markedly higher short-wavelength intensity and greater total spectral area, whereas pathology-negative nodes showed a higher I630/I360 ratio, higher intensity at 700 nm, a deeper local blue trough, and a larger red shoulder. This bidirectional spectral pattern may help explain why the hybrid model had a higher discrimination point estimate than the simpler candidate models. However, model-family comparisons were exploratory and were not designed to establish formal superiority. Possible contributors to the observed wavelength-dependent differences include changes in superficial scattering, cellular density, chromophore absorption, stromal architecture, and vascular content. This study did not include site-matched quantitative optical-histological measurements capable of establishing these mechanisms. These explanations are therefore hypothesis generating and should not be interpreted as demonstrated biological causes of the spectral differences.

This study extends, but is independent of, the earlier ESS study of cervical lymph nodes [14]. The previously published study examined 347 spectra obtained from 130 formalin-fixed lymph nodes in 13 patients and reported high sensitivity but lower specificity using selected wavelength intensities and a simple cutoff. The present cohort comprised fresh lymph nodes from 45 different patients. No participant, lymph node, raw spectrum, pathology outcome, or derived model output from the earlier dataset was included in this analytical dataset or used for model fitting, hyperparameter tuning, probability calibration, threshold selection, or performance estimation. All data-derived spectral and performance figures in this report were generated from the present cohort rather than from the previously published dataset. This analysis also differed by using 3 spatially separated acquisitions per node, explicit quality control, node-level probability aggregation, and internal validation grouped by patient.

The direction of some individual spectral features differed between the 2 studies. The earlier study found generally greater scattering intensity in pathology-positive measurements, including at longer wavelengths, whereas the present node-level analysis found higher I700 intensity, a higher I630/I360 ratio, a deeper blue trough, and a larger red shoulder in pathology-negative nodes. The differing wavelength-specific directions should not be interpreted as direct replication or contradiction because the studies differed materially in specimen state, acquisition context, analytical unit, preprocessing, aggregation, and modeling. The earlier specimens were formalin fixed and analyzed at individual-measurement level, whereas the present specimens were assessed fresh and spectral information was aggregated within lymph nodes. Taken together, the studies support the broader hypothesis that metastatic involvement is associated with differences in optical scattering profiles, while the wavelength-specific direction and magnitude of those differences require independent investigation under standardized acquisition conditions.

Repeated acquisition reduced dependence on a single contact site but did not remove technical variation. Within-node ICCs ranged from 0.78 for blue trough depth to 0.91 for I630 intensity, indicating that amplitude-related features were more repeatable than some local shape features. Operator-specific AUROCs were similar, but each node was assessed by only 1 operator, so a direct paired estimate of interoperator reproducibility was not possible. Future studies should use locked surface-preparation and timing procedures, record probe pressure and contact angle prospectively, and include repeated assessments of the same nodes by multiple operators.

This observation may also have a biological explanation. The ESS probe interrogates only a small superficial tissue volume, approximately 500×300×300 µm, meaning that each spectrum represents a local optical microsample rather than the entire node. Metastatic deposits may be focal, peripheral, central, or microscopic, and individual spectra may therefore capture slightly different aspects of nodal architecture. Aggregating multiple spectra from the same lymph node is consequently more than a statistical exercise; it represents repeated sampling of biological heterogeneity. The improvement observed with node-level aggregation suggests that repeated optical interrogation stabilizes classification by integrating information across multiple microenvironments within the same node. This interpretation is consistent with the probe geometry described in the original ESS nodal study, where collected photons originated from a defined superficial scattering volume rather than a whole-node measurement.

This node-level framing is particularly relevant when compared with imaging. Clinical examination, CT, MRI, PET-CT, and ultrasound remain essential in preoperative staging, yet each can struggle with small-volume or biologically ambiguous nodal disease. Historical clinical and radiological studies have shown substantial false-negative and false-positive rates in the neck, and contemporary meta-analyses continue to show that advanced imaging is useful but imperfect [6,7,22,23]. ESS addresses a different point in the pathway. It is not a preoperative staging test. Its most realistic role is ex vivo assessment of dissected lymph nodes, helping to identify nodes that warrant closer histopathological attention, expedited review, or targeted deeper sectioning.

Patient-level sensitivity was 96% and specificity was 90% when a patient was classified as positive if at least 1 ESS-assessed node exceeded the cross-validation–derived summary threshold. This analysis provides an exploratory indication of performance at patient level, but it was based only on the ESS-assessed node set and should not be interpreted as complete pathological staging of the neck. The maximum-node approach may also be influenced by the number of nodes assessed per patient because patients contributing more nodes have more opportunities to generate a high node-level probability. Prospective evaluation in which the clinically relevant nodal yield is assessed according to a predefined sampling protocol will be required to establish patient-level clinical performance.

The potential clinical role of ESS should be framed as augmentation rather than replacement. Histopathology remains the reference standard and must continue to determine the final diagnosis. ESS may warrant prospective evaluation as an investigational triage tool for identifying high-probability nodes that could be prioritized for review or additional pathological assessment, while all nodes continue to undergo the required routine histopathological pathway. Whether this approach improves turnaround time, workload, or diagnostic safety remains to be demonstrated prospectively.

Any future clinical system would need to provide automated quality control and a simple probability or indeterminate output within a time compatible with gross specimen assessment. Evaluation should compare routine pathology with and without ESS-guided specimen prioritization and, where relevant, benchmark diagnostic performance and turnaround time against rapid sentinel-node or frozen-section pathways [24].

The observed node-level NPV of 96.6% at the cross-validation–derived summary threshold is potentially relevant to future triage applications but remains dependent on disease prevalence and should be interpreted as a secondary descriptive operating characteristic of this internally validated cohort. The prevalence of metastatic nodes will differ between elective and therapeutic neck dissections, tumor stages, anatomical sites, sampling strategies, and referral settings. Calibration was acceptable within the present cohort, but both predictive values and probability calibration may change in an independent population. External validation should therefore use a prospectively locked threshold, evaluate whether recalibration is required, and consider an indeterminate probability range rather than assuming that every node should receive a forced binary classification.

### Strengths

The study has several strengths. Histopathology was used as the reference standard, repeated spectra were acquired from each node, and explicit quality-control criteria were applied before modeling. The primary validation kept all nodes and spectra from each patient together, and model fitting, hyperparameter selection, probability calibration where required, and threshold optimization were restricted to training data within the nested validation process. Performance was reported with patient-clustered CIs, calibration measures, patient-level results, case-level error review, and technical repeatability analyses. Moreover, 6 node-level index-test no-results were also reported explicitly rather than being treated as model classifications. The lower discrimination of area-normalized and shape-focused analyses suggests that amplitude information contributed materially to model performance in this cohort, although this finding reinforces the need for careful control of acquisition conditions and independent external validation.

### Limitations

Several limitations remain. First, this was a single-center model-development and internal-validation study involving 45 patients. Although the analytical dataset contained 274 analyzable nodes and 760 retained spectra, observations were clustered within patients; the effective number of independent biological units was substantially smaller than the number of spectra or nodes, and spectrum-level fitting gave greater influence to patients contributing more nodes and to nodes retaining 3 rather than 2 spectra. Future work should compare patient- or node-weighted fitting with direct node-level spectral aggregation. Prospective external validation is, therefore, required. Second, the patient-level analysis represented the ESS-assessed node set rather than complete pathological staging of every node recovered from each neck dissection. The ESS-assessed nodes represented a pragmatic availability-based sample rather than the complete pathological nodal yield, so the observed node-level prevalence and diagnostic performance may not transfer directly to an unselected routine pathology population. Third, 6 of 280 ESS-assessed nodes produced no analyzable node-level results, and all 6 were pathology negative. The small number of no-results precluded reliable assessment of whether acquisition failure was associated with pathology, node characteristics, or other technical factors. Fourth, deeper levels and pancytokeratin immunohistochemistry were used selectively rather than systematically, so very small metastatic deposits could theoretically have been missed by the reference-standard assessment. Fifth, some technical variables were derived from archival records, while probe pressure and precise contact angle were not measured quantitatively. Sixth, each lymph node was assessed by only 1 operator, preventing paired assessment of interoperator reproducibility. Operator, acquisition batch, and case mix were not fully crossed within the same specimens, so operator-stratified and batch-based analyses could not completely separate technical effects from differences in patient or nodal characteristics. Seventh, amplitude contributed materially to discrimination and may be influenced by surface hydration, blood contamination, tissue handling, probe contact, system response, and elapsed time from excision. Eighth, the hybrid model family was selected because it had the highest pooled patient-grouped out-of-fold AUROC point estimate among the candidate families. Its AUROC of 0.966 is therefore an internally validated, exploratory estimate of the selected model’s performance, rather than independent validation of a prospectively fixed final model. Using the same out-of-fold predictions for model-family selection and performance reporting may introduce selection optimism; independent evaluation of a prospectively specified and locked model remains necessary. Ninth, the pooled binary operating characteristics were calculated using a summary threshold derived from the distribution of training-derived thresholds after repeated internal cross-validation and should be interpreted as descriptive rather than as validation of a prospectively locked clinical threshold. Tenth, the specimens were collected between 2016 and 2021 using a research ESS platform, so differences in contemporary devices, specimen handling, staging practice, and pathology workflows may affect transportability. Finally, 3 fixed acquisitions sampled only small superficial tissue volumes and may not consistently capture focal, peripheral, or heterogeneous metastatic deposits. Prospective studies should evaluate predefined acquisition protocols, denser or scanned sampling, automated quality control, and an independently locked model and decision threshold.

### Future Work

A particularly important next step will be site-matched optical-pathological correlation. Future studies could coregister ESS acquisition points with digital histopathology and quantitative image analysis to determine whether wavelength-specific changes correlate with tumor area, nuclear density, keratinization, fibrosis, inflammatory infiltrates, vascularity, or other measurable tissue features. Such work would allow ESS findings to move beyond diagnostic discrimination towards biologically interpretable optical pathology and may clarify the cellular and subcellular origins of the wavelength patterns identified in this study.

Future evaluation should use a prospective multicenter design in which the acquisition protocol, preprocessing sequence, quality-control rules, classifier, probability-calibration procedure, and decision threshold are specified and locked before evaluation of the external validation cohort. The study should include multiple devices, operators, pathology teams, and clinically relevant patient groups. Outcomes should include discrimination, calibration, operator reproducibility, acquisition failure, indeterminate results, turnaround time, pathology workload, missed disease, and costs. The appropriate comparison is not ESS vs histopathology as competing diagnostic methods, but routine pathology with and without ESS-guided specimen prioritization.

### Conclusion

In this single-center ex vivo model development and internal validation study, ESS showed promising node-level discrimination of metastatic cervical lymph nodes after repeated spectral acquisition and node-level aggregation. Patient-grouped validation produced lower performance estimates than validation grouped only at lymph-node level, emphasizing the importance of accounting for clustering of multiple nodes within patients. The findings remain exploratory and do not establish clinical utility. ESS should therefore be regarded as an investigational adjunct for pathology triage rather than a clinically ready diagnostic test. Prospective multicenter external validation using a prespecified and locked analytical pipeline and decision threshold, together with explicit assessment of no-results, calibration, paired operator reproducibility, acquisition variability, and workflow performance, is required before clinical implementation.

## Supplementary material

10.2196/103960 Multimedia Appendix 1 Supplementary tables and figures.

10.2196/103960 Checklist 1 TRIPOD+AI completed checklist.

10.2196/103960 Checklist 2 STARD-AI completed checklist.

10.2196/103960 Checklist 3 STARD 2015 completed checklist.

## Abbreviations

AUROC: area under the receiver operating characteristic curve
CT: computed tomography
ESS: elastic scattering spectroscopy
ICC: intraclass correlation coefficient
MRI: magnetic resonance imaging
NPV: negative predictive value
PET-CT: positron emission tomography-computed tomography
PPV: positive predictive value
SNV: standard normal variate
STARD: Standards for Reporting of Diagnostic Accuracy Studies
TRIPOD: Transparent Reporting of a Multivariable Prediction Model for Individual Prognosis or Diagnosis

## References

[1] Bray F, Laversanne M, Sung H (2024). Global cancer statistics 2022: GLOBOCAN estimates of incidence and mortality worldwide for 36 cancers in 185 countries. CA Cancer J Clin.

[2] Barsouk A, Aluru JS, Rawla P, Saginala K, Barsouk A (2023). Epidemiology, risk factors, and prevention of head and neck squamous cell carcinoma. Med Sci (Basel).

[3] Dunn LA, Ho AL, Pfister DG (2026). Head and neck cancer: a review. JAMA.

[4] Alqutub S, Alqutub A, Bakhshwin A (2024). Histopathological predictors of lymph node metastasis in oral cavity squamous cell carcinoma: a systematic review and meta-analysis. Front Oncol.

[5] Yu YF, Cao LM, Li ZZ (2025). Frequency of lymph node metastases at different neck levels in patients with oral squamous cell carcinoma: a systematic review and meta-analysis. Int J Surg.

[6] Alsibani A, Alqahtani A, Almohammadi R (2024). Comparing the efficacy of CT, MRI, PET-CT, and US in the detection of cervical lymph node metastases in head and neck squamous cell carcinoma with clinically negative neck lymph node: a systematic review and meta-analysis. J Clin Med.

[7] Guedj D, Neveü S, Becker M, Mermod M (2024). FDG PET-CT for the detection of occult nodal metastases in head and neck cancer: a systematic review and meta-analysis. Cancers (Basel).

[8] Jiang S, Locatello LG, Maggiore G, Gallo O (2023). Radiomics-based analysis in the prediction of occult lymph node metastases in patients with oral cancer: a systematic review. J Clin Med.

[9] Khong B, Ferlito S, Quek S (2024). Past, present, and future diagnostic methods for the early noninvasive detection of oral premalignant lesions: a state of the art and systematic review. Ear Nose Throat J.

[10] Zhang Y, Li Z, Zhang C (2025). Recent advances of photodiagnosis and treatment for head and neck squamous cell carcinoma. Neoplasia.

[11] Hanna K, Asiedu AL, Theurer T (2024). Advances in Raman spectroscopy for characterising oral cancer and oral potentially malignant disorders. Expert Rev Mol Med.

[12] Wu IC, Chen YC, Karmakar R (2024). Advancements in hyperspectral imaging and computer-aided diagnostic methods for the enhanced detection and diagnosis of head and neck cancer. Biomedicines.

[13] Alajaji SA, Sabzian R, Wang Y, Sultan AS, Wang R (2025). A scoping review of infrared spectroscopy and machine learning methods for head and neck precancer and cancer diagnosis and prognosis. Cancers (Basel).

[14] Jerjes W, Swinson B, Pickard D, Thomas GJ, Hopper C (2004). Detection of cervical intranodal metastasis in oral cancer using elastic scattering spectroscopy. Oral Oncol.

[15] Bigio IJ, Mourant JR (1997). Ultraviolet and visible spectroscopies for tissue diagnostics: fluorescence spectroscopy and elastic-scattering spectroscopy. Phys Med Biol.

[16] Bigio IJ, Bown SG, Briggs G (2000). Diagnosis of breast cancer using elastic-scattering spectroscopy: preliminary clinical results. J Biomed Opt.

[17] Collins GS, Moons KGM, Dhiman P (2024). TRIPOD+AI statement: updated guidance for reporting clinical prediction models that use regression or machine learning methods. BMJ.

[18] Sounderajah V, Guni A, Liu X (2025). The STARD-AI reporting guideline for diagnostic accuracy studies using artificial intelligence. Nat Med.

[19] Moons KGM, Damen JAA, Kaul T (2025). PROBAST+AI: an updated quality, risk of bias, and applicability assessment tool for prediction models using regression or artificial intelligence methods. BMJ.

[20] Vasey B, Nagendran M, Campbell B (2022). Reporting guideline for the early stage clinical evaluation of decision support systems driven by artificial intelligence: DECIDE-AI. BMJ.

[21] Bossuyt PM, Reitsma JB, Bruns DE (2015). STARD 2015: an updated list of essential items for reporting diagnostic accuracy studies. BMJ.

[22] Woolgar JA, Vaughan ED, Scott J, Brown JS (1994). Pathological findings in clinically false-negative and false-positive neck dissections for oral carcinoma. Ann R Coll Surg Engl.

[23] Wide JM, White DW, Woolgar JA, Brown JS, Vaughan ED, Lewis-Jones HG (1999). Magnetic resonance imaging in the assessment of cervical nodal metastasis in oral squamous cell carcinoma. Clin Radiol.

[24] Abukhder M, Sahovaler A, Vrakas P, McGurk M, Thavaraj S, Schilling C (2024). Frozen section analysis of sentinel nodes in patients with oral squamous cell carcinoma: a systematic review and meta-analysis. JAMA Otolaryngol Head Neck Surg.

