# Supplementary material for: Detection of Cervical Nodal Metastasis in Oral Squamous Cell Carcinoma Using Elastic Scattering Spectroscopy: Ex Vivo Diagnostic Modeling Study
Source: JMIR Cancer. 2026 Oct 13;12:e103960. doi: 10.2196/103960 (PMC13661036; doi:10.2196/103960)
Supplement: Supplementary file 1 [file cancer-v12-e103960-s001.docx]

Multimedia Appendix Table S1. Analytical workflow and model specification

| Component | Specification |
| --- | --- |
| Primary validation | StratifiedGroupKFold, 5 outer folds × 10 repetitions; groups=patient ID; pathology-class stratification; shuffle=True; repetition-specific random_state=202601–202610 |
| Inner validation | StratifiedGroupKFold, 4 folds within each outer training set; groups=patient ID; pathology-class stratification; shuffle=True; corresponding repetition-specific random state |
| Analysis wavelength window | 350–850 nm |
| Smoothing | Savitzky–Golay filter; second-order polynomial; 11-point window |
| Amplitude branch | Eight prespecified optical features extracted from Savitzky–Golay-smoothed raw-amplitude spectra; robust scaling fitted within inner training folds |
| Shape branch | Standard normal variate normalisation followed by principal component analysis fitted within inner training folds |
| Candidate principal-component counts | 10, 20, 30, and 40 |
| Baseline I630 rule | Median I630 intensity across retained spectra within each lymph node; threshold selected by maximising the Youden index using patient-grouped inner-validation predictions within each outer training set; threshold then applied unchanged to the corresponding held-out outer-test patients |
| Selected hybrid model family | Elastic-net logistic regression combining selected principal-component scores from the shape branch with the 8 robustly scaled optical features from the amplitude branch; the hybrid model family was selected for detailed analysis through exploratory comparison of pooled patient-grouped out-of-fold ROC AUC estimates |
| Regularisation grid | C values 0.01, 0.03, 0.1, 0.3, 1, 3, and 10; l1 ratios 0.1, 0.3, 0.5, 0.7, and 0.9 |
| Selected principal-component count | Modal selected value across the 50 outer fits: 20; range: 10–30 |
| Selected C | Modal selected value: 0.3; range across outer fits: 0.1–1 |
| Selected l1 ratio | Modal selected value: 0.5; range across outer fits: 0.3–0.7 |
| Selected class weighting | Balanced in 34/50 outer fits; none in 16/50 |
| Logistic-regression implementation | Solver: saga; maximum iterations: 5000 |
| Class weighting | None or balanced; selected in the inner folds |
| Linear SVM probabilities | Platt scaling fitted exclusively within inner training folds |
| Model-selection criterion | Mean patient-grouped inner-fold ROC AUC was used to select configurations and hyperparameters within each candidate model family. The hybrid model family was selected for detailed analysis because it had the highest pooled patient-grouped out-of-fold ROC AUC point estimate. Between-family selection and performance reporting were exploratory and used the same out-of-fold predictions |
| Node aggregation | Mean probability across retained spectra within each node; repeated outer-fold node probabilities were averaged across the 10 repetitions |
| Single-spectrum sensitivity analysis | First quality-control-passing spectrum from each lymph node according to recorded acquisition order; selection was independent of histopathological status and model output |
| Two-spectrum sensitivity analysis | First 2 quality-control-passing spectra from each lymph node according to recorded acquisition order; selection was independent of histopathological status and model output |
| Spectral-quality sensitivity analysis | Retained spectra were ranked by signal-to-noise ratio, defined as the mean spectral intensity across 350–850 nm divided by the standard deviation of the residual between the raw and Savitzky–Golay-smoothed spectrum. Within each outer training set, the 10th percentile of the signal-to-noise-ratio distribution defined the exclusion threshold, which was applied unchanged to the corresponding outer test set; histopathological status and model output were not used to define spectral quality |
| Threshold selection | Youden index applied to patient-grouped inner-validation predictions within each outer training set. Threshold optimisation therefore used training data only. The 50 training-derived thresholds had a median of 0.38 (IQR 0.35–0.41). For pooled descriptive operating characteristics, this median summary threshold was applied once to each node’s averaged patient-grouped held-out probability; no pooled outcome or outer-test outcome was used to select or modify the threshold |
| Patient-level output | Positive when at least 1 ESS-assessed node exceeded the cross-validation-derived summary node-level threshold; interpreted as an exploratory classification of the assessed node set rather than complete pathological neck staging |
| Calibration assessment | Brier score, calibration-in-the-large, calibration slope, and a grouped calibration plot from averaged patient-grouped out-of-fold node probabilities. The plot compares the observed pathology-positive proportion with the mean predicted probability within probability groups that were not constrained to contain equal numbers of nodes |
| Confidence intervals | 2000 patient-clustered bootstrap resamples retaining all lymph nodes belonging to each resampled patient |
| Within-node agreement | One-way random-effects, absolute-agreement, single-measure intraclass correlation coefficient (ICC[1,1]); 95% confidence intervals estimated from the corresponding F distribution using SciPy 1.15.0 |
| Software | Python 3.12.8; NumPy 2.2.1; pandas 2.2.3; SciPy 1.15.0; scikit-learn 1.6.0 |
| Random seeds | 202601–202610 for the 10 outer repetitions |

All preprocessing, dimensionality reduction, scaling, model fitting, hyperparameter tuning, probability calibration where required, and threshold optimisation were performed using training data within the nested patient-grouped validation process. Outer test patients were used only to generate held-out predictions. Model-family comparisons were exploratory. The pooled summary threshold used for descriptive binary operating characteristics was not tuned or modified using pooled or outer-test outcomes.

Multimedia Appendix Table S2. Case-level review of misclassified lymph nodes

| Node ID | Error | Node size, mm | Metastatic deposit | Neck level | Histopathological context | Out-of-fold probability |
| --- | --- | --- | --- | --- | --- | --- |
| P03-N01 | False negative | 6 | 0.4-mm micrometastasis | II | Mild sinus histiocytosis; no necrosis, keratinisation, or extranodal extension | 0.197 |
| P07-N01 | False negative | 8 | 0.8-mm micrometastasis | I | No substantial reactive change; no necrosis, keratinisation, or extranodal extension | 0.142 |
| P11-N01 | False negative | 11 | 2.3-mm peripheral macrometastasis | III | Focal keratinisation; no necrosis or extranodal extension | 0.028 |
| P16-N01 | False negative | 5 | 0.3-mm micrometastasis | II | Moderate follicular hyperplasia; no necrosis, keratinisation, or extranodal extension | 0.053 |
| P21-N01 | False negative | 9 | 1.6-mm micrometastasis | IV | No substantial reactive change; no necrosis, keratinisation, or extranodal extension | 0.239 |
| P25-N01 | False negative | 13 | 2.8-mm peripheral macrometastasis | I | Focal tumour necrosis; no keratinisation or extranodal extension | 0.083 |
| P31-N01 | False positive | 18 | No metastasis | II | Marked follicular hyperplasia; no necrosis or keratinisation | 0.995 |
| P31-N02 | False positive | 14 | No metastasis | II | Prominent sinus histiocytosis; no necrosis or keratinisation | 0.869 |
| P31-N03 | False positive | 22 | No metastasis | III | Paracortical hyperplasia; no necrosis or keratinisation | 0.420 |
| P31-N04 | False positive | 12 | No metastasis | I | Marked vascular congestion; no necrosis or keratinisation | 0.949 |
| P42-N01 | False positive | 20 | No metastasis | II | Suppurative inflammation with focal necrosis; no keratinisation | 0.710 |
| P42-N02 | False positive | 16 | No metastasis | III | Prominent sinus histiocytosis; no necrosis or keratinisation | 0.976 |
| P42-N03 | False positive | 9 | No metastasis | I | Surface haemorrhage and vascular congestion; no necrosis or keratinisation | 0.934 |
| P42-N04 | False positive | 17 | No metastasis | IV | Reactive follicular hyperplasia; no necrosis or keratinisation | 0.896 |

Misclassification status was determined using the cross-validation-derived summary node-level threshold of 0.38. The 6 pathology-negative node-level index-test no-results are not included in this misclassification table. Node identifiers are deidentified analytical identifiers.

Multimedia Appendix Table S3. Within-node agreement and technical variability analyses

| Variable | Analysis | Result |
| --- | --- | --- |
| I630 intensity | Within-node agreement across spatially separated acquisitions | ICC 0.91 (95% CI 0.88–0.93); median coefficient of variation 7.4% |
| Total spectral area | Within-node agreement across spatially separated acquisitions | ICC 0.89 (95% CI 0.86–0.92); median coefficient of variation 8.1% |
| Red shoulder height | Within-node agreement across spatially separated acquisitions | ICC 0.84 (95% CI 0.80–0.88); median coefficient of variation 10.3% |
| Blue trough depth | Within-node agreement across spatially separated acquisitions | ICC 0.78 (95% CI 0.72–0.83); median coefficient of variation 12.0% |
| Spectrum-level hybrid probability | Within-node agreement across spatially separated acquisitions | ICC 0.87 (95% CI 0.83–0.90); median coefficient of variation 9.6% |
| Operator A | Operator-stratified discrimination | Patient-grouped ROC AUC 0.968 (95% CI 0.940–0.985) |
| Operator B | Operator-stratified discrimination | Patient-grouped ROC AUC 0.961 (95% CI 0.918–0.985) |
| Operator difference | Operator-stratified discrimination | ROC AUC difference 0.007 (95% CI −0.031 to 0.044) |
| Acquisition within 30 minutes | Timing sensitivity analysis | Patient-grouped ROC AUC 0.967 (95% CI 0.936–0.985) |
| Acquisition after 30 minutes | Timing sensitivity analysis | Patient-grouped ROC AUC 0.958 (95% CI 0.910–0.983) |
| Leave-one-patient-out validation | Patient-grouped robustness analysis | ROC AUC 0.961 (95% CI 0.927–0.982) |
| Acquisition batch | Leave-one-batch-out validation | ROC AUC 0.952 (95% CI 0.909–0.979) |

AUC: area under the receiver operating characteristic curve; CI: confidence interval; ICC: intraclass correlation coefficient. Each node was acquired by 1 operator, so operator-specific estimates compare independent operator-specific case series rather than paired measurements of the same node and do not constitute a direct estimate of interoperator reproducibility. Timing, operator, and batch analyses were treated as sensitivity analyses because technical factors were not fully crossed within the same specimens. Ninety-five percent confidence intervals for ROC AUC estimates were estimated using patient-clustered bootstrap resampling, retaining all lymph nodes belonging to each resampled patient.

**Multimedia Appendix Figure S1.** Leakage-controlled patient-grouped internal-validation workflow. Repeated nested 5-fold internal validation used patient ID as the grouping variable, with 10 outer repetitions. Every spectrum and lymph node from each patient remained in the same outer fold, and inner validation within each outer training set was also grouped by patient. Preprocessing, spectral representation, hyperparameter tuning, probability calibration where required, and decision-threshold optimisation were undertaken using training data only. The resulting pipeline was then applied to the corresponding held-out patients to generate spectrum-level probabilities, which were aggregated within lymph nodes. Each node therefore received 1 held-out probability in each of 10 repetitions, and these probabilities were averaged for ROC and calibration analyses. The 50 training-derived thresholds had a median of 0.38 (IQR 0.35–0.41). This median summary threshold was applied to averaged held-out probabilities only to generate secondary descriptive binary operating characteristics; no pooled outcome or outer-test outcome was used to select or modify the threshold. The maximum averaged node probability within each patient was used for patient-level ROC analysis.

**Multimedia Appendix Figure S2.** Technical repeatability and robustness. Panel A shows within-node intraclass correlation coefficients and 95% confidence intervals for I630 intensity, total spectral area, hybrid probability, red-shoulder height, and blue-trough depth. Panel B shows patient-grouped ROC AUC estimates for operator groups, acquisition timing, leave-one-patient-out validation, and leave-one-batch-out validation. Numerical ROC AUC estimates and their 95% confidence intervals for all analyses in Panel B are reported in Multimedia Appendix Table S3. Each lymph node was assessed by 1 operator, so the operator comparison represents independent operator-specific case series rather than paired assessment of the same nodes and should not be interpreted as direct interoperator reproducibility.
