# Supplementary material for: Detection of Cervical Nodal Metastasis in Oral Squamous Cell Carcinoma Using Elastic Scattering Spectroscopy: Ex Vivo Diagnostic Modeling Study
Source: JMIR Cancer. 2026 Oct 13;12:e103960. doi: 10.2196/103960 (PMC13661036; doi:10.2196/103960)
Supplement: Supplementary file 2 [file cancer-v12-e103960-s002.docx]

# TRIPOD+AI completed checklist

*Source: Collins GS, Moons KGM, Dhiman P, et al. TRIPOD+AI statement. BMJ. 2024;385:e078378. doi:10.1136/bmj-2023-078378.*

| **Section / item** | **Checklist recommendation** | **Status** | **Reported in manuscript / comment** |
| --- | --- | --- | --- |
| **Title 1 (D;E)** | Identify the study as developing or evaluating a multivariable prediction model, the target population, and the outcome to be predicted. | **Reported** | p1; title identifies an ex vivo diagnostic modelling study for cervical nodal metastasis in oral squamous cell carcinoma. |
| **Abstract 2 (D;E)** | Report the study using the TRIPOD+AI for Abstracts checklist. | **Reported** | pp1-2; structured Background, Objective, Methods, Results, and Conclusions. |
| **Background 3a (D;E)** | Explain the diagnostic/prognostic healthcare context and the rationale for developing or evaluating the model, including existing models. | **Reported** | pp2-3; Introduction describes the clinical pathway, limitations of current assessment, prior ESS work, and rationale. |
| **Background 3b (D;E)** | Describe the target population, intended purpose in the care pathway, and intended users. | **Reported** | pp2-3 and pp14-16; adults with oral squamous cell carcinoma; investigational ex vivo pathology-triage adjunct. |
| **Background 3c (D;E)** | Describe known health inequalities between sociodemographic groups. | **Not specifically assessed** | No dedicated inequality analysis; age and sex are reported in Table 1 and generalisability is discussed on pp14-15. |
| **Objectives 4 (D;E)** | Specify the study objectives and whether the study concerns model development, evaluation, or both. | **Reported** | p3; objective is development and internal evaluation of node-level ESS classification. |
| **Data 5a (D;E)** | Describe data sources for development and evaluation, the rationale for their use, and representativeness. | **Reported** | pp3-4 and p8; single-centre observational cohort with fresh ESS measurements, archived spectra, and linked histopathology; representativeness limitations stated. |
| **Data 5b (D;E)** | Specify participant-data dates, including accrual and follow-up where applicable. | **Reported** | pp3-4 and Table 2 (p21); collection September 2016-March 2021, curation in 2025, modelling January-May 2026. |
| **Participants 6a (D;E)** | Specify key elements of the setting, including number and location of centres. | **Reported** | pp3-4; single centre at University College London Hospitals. |
| **Participants 6b (D;E)** | Describe participant eligibility criteria. | **Reported** | p4; inclusion and exclusion criteria are stated. |
| **Participants 6c (D;E)** | Describe treatments received and how they were handled, if relevant. | **N/A** | Ex vivo diagnostic study; surgery and neck dissection were clinical care, not study-assigned treatment. |
| **Data preparation 7 (D;E)** | Describe data preprocessing and quality checking, including consistency across relevant sociodemographic groups. | **Reported** | pp5-6 and Multimedia Appendix Table S1 (pp33-34); common QC and preprocessing rules were applied to all spectra. |
| **Outcome 8a (D;E)** | Define the predicted outcome, time horizon, assessment method and rationale, including consistency across demographic groups. | **Reported** | p5; contemporaneous lymph-node histopathology defined metastatic status; no prognostic time horizon applies. |
| **Outcome 8b (D;E)** | If outcome assessment is subjective, describe outcome-assessor qualifications and demographic characteristics. | **Partial** | p5; consultant head-and-neck pathologists are described, but assessor demographics are not reported. |
| **Outcome 8c (D;E)** | Report blinding of outcome assessment. | **Reported** | p5; pathologists were blinded to ESS spectra, probabilities, and model outputs. |
| **Predictors 9a (D)** | Describe the choice and any preselection of initial predictors. | **Reported** | p6; eight optical features were specified from interpretability and prior ESS work; full-spectrum representations were also evaluated. |
| **Predictors 9b (D;E)** | Define all predictors, including how and when they were measured and any blinding. | **Reported** | pp4-6; acquisition protocol, wavelength window, feature definitions, node aggregation, and pathology-blinded QC are specified. |
| **Predictors 9c (D;E)** | If predictor measurement is subjective, describe predictor-assessor qualifications and demographic characteristics. | **Partial** | p4; two trained operators are described, but operator demographics are not reported. |
| **Sample size 10 (D;E)** | Explain how study size was determined and justify adequacy, including calculations where performed. | **Reported** | p8; no formal calculation was undertaken; the complete eligible linked cohort was analysed and this is acknowledged as a limitation. |
| **Missing data 11 (D;E)** | Describe handling of missing data and reasons for omissions. | **Reported** | pp5-6, Table 3 (p22), and Figure 3 (p28); 80 spectra were excluded by prespecified QC and six node-level no-results were reported separately. |
| **Analysis 12a (D)** | Describe how data were used for model development and performance evaluation, including partitioning. | **Reported** | pp6-8 and Table S1; repeated nested patient-grouped cross-validation with 50 outer splits. |
| **Analysis 12b (D)** | Describe predictor handling, including functional form, rescaling, transformation, or standardisation. | **Reported** | pp6-8 and Table S1; smoothing, robust scaling, SNV, PCA, and node aggregation are specified. |
| **Analysis 12c (D)** | Specify model type, rationale, all model-building steps, hyperparameter tuning, and internal validation. | **Reported** | pp6-8 and Table S1 (pp33-34); candidate models, grids, fitting, calibration, tuning, and validation are detailed. |
| **Analysis 12d (D;E)** | Describe how heterogeneity across clusters was handled and quantified. | **Reported** | pp6-8; patient grouping prevented leakage. Operator, timing, batch, and leave-one-patient-out analyses are reported on p11 and in Table S3. |
| **Analysis 12e (D;E)** | Specify measures and plots used to evaluate model performance and compare models. | **Reported** | pp7-8; discrimination, calibration, threshold metrics, CIs, ROC/calibration plots, and exploratory model comparisons are specified. |
| **Analysis 12f (E)** | Describe model updating arising from evaluation. | **N/A** | No model updating or recalibration on an external dataset was performed. |
| **Analysis 12g (E)** | For model evaluation, describe how predictions were calculated. | **Reported** | pp7-8 and Table S1; held-out spectrum probabilities were generated by the locked fold-specific pipeline and averaged within nodes and repetitions. |
| **Class imbalance 13 (D;E)** | If class-imbalance methods were used, explain why and how, including any recalibration. | **Reported** | p6 and Table S1; none versus balanced class weighting was selected within inner folds; calibration was assessed separately. |
| **Fairness 14 (D;E)** | Describe methods used to address model fairness and their rationale. | **Not undertaken** | No formal fairness adjustment or demographic subgroup performance analysis was undertaken; the small single-centre cohort and transportability limitations are discussed on pp14-15. |
| **Model output 15 (D)** | Specify model output and the rationale and derivation of any classification threshold. | **Reported** | p7 and Table S1; node-level probability output, training-only Youden thresholds, and pooled descriptive threshold are explained. |
| **Development vs evaluation 16 (D;E)** | Identify differences between development and evaluation data in setting, eligibility, outcome, and predictors. | **N/A** | No independent external dataset; development and internal evaluation used patient-grouped resampling from the same source cohort. The node-grouped comparison is reported on p10. |
| **Ethical approval 17 (D;E)** | Name the ethics committee and describe consent or waiver. | **Reported** | Materials and methods—Ethics Approval reports the REC application submission date of 26 April 2006, the favourable-opinion letter dated 31 July 2006, the approving committee and reference 06/Q0505/39, and the scope described in the application and protocol. The provisions for annual local organisational governance checks and the Chief Investigator’s responsibilities are described, together with written informed consent, deidentification, restricted data access, and absence of participant compensation. |
| **Funding 18a (D;E)** | Give funding source and funder role. | **Reported** | p16; no financial support was received. |
| **Conflicts 18b (D;E)** | Declare conflicts of interest and financial disclosures. | **Reported** | p16; none declared. |
| **Protocol 18c (D;E)** | State where the study protocol can be accessed or that no protocol was prepared. | **Partial** | p8 describes the analysis plan and states that the exploratory modelling plan was not prospectively registered; no separate protocol location is given. |
| **Registration 18d (D;E)** | Provide registration details or state that the study was not registered. | **Reported** | p8; the modelling analysis was not prospectively registered. |
| **Data sharing 18e (D;E)** | Describe data availability. | **Reported** | p16; deidentified dataset and data dictionary may be available on reasonable request subject to governance. |
| **Code sharing 18f (D;E)** | Describe analytical-code availability. | **Reported** | p16 and Table S1; code may be available on reasonable request and the analytical specification is reported. |
| **PPI 19 (D;E)** | Describe patient and public involvement or state that none occurred. | **Not reported** | No patient or public involvement statement is included in the manuscript. |
| **Participants 20a (D;E)** | Describe participant flow, outcome counts, and follow-up where applicable; a diagram may be used. | **Reported** | p9 and Figure 3 (p28); 52 assessed, 45 included, 280 nodes, QC flow, class counts, and no-results. |
| **Participants 20b (D;E)** | Report characteristics by data source/setting, including dates, predictors, demographics, sample size, outcome events and missing data. | **Reported** | p9 and Tables 1-3 (pp20-22). |
| **Participants 20c (E)** | For evaluation, compare important predictor and outcome distributions with development data. | **N/A** | No fixed independent evaluation dataset; repeated internal cross-validation used the same source cohort. |
| **Model development 21 (D;E)** | State participant and outcome-event numbers in each analysis. | **Reported** | pp1, 9-11 and Tables 2, 3, 5, and 6; 45 patients, 274 analysable nodes, 96 positive nodes, and 760 spectra. |
| **Model specification 22 (D)** | Provide the full model or implementation details sufficient for prediction and third-party evaluation, including access restrictions. | **Partial** | Table S1 reports the complete analytical workflow and hyperparameter ranges; the fitted model object is not publicly deposited, but code may be requested under governance. |
| **Performance 23a (D;E)** | Report model performance with confidence intervals, including key subgroups where relevant, and consider plots. | **Reported** | pp10-11; Tables 5-6, Figures 6-7, Table S3, and Figure S2 report performance, CIs, calibration, and technical subgroups. |
| **Performance 23b (D;E)** | If examined, report heterogeneity in performance across clusters. | **Reported** | p11, Table S3, and Figure S2; operator, timing, patient, and batch sensitivity analyses. |
| **Model updating 24 (E)** | Report any model updating and subsequent performance. | **N/A** | No model updating was undertaken. |
| **Interpretation 25 (D;E)** | Interpret results, including fairness, in relation to objectives and previous studies. | **Reported** | pp12-16; findings are compared with prior ESS work, and interpretation is explicitly cautious and hypothesis-generating. |
| **Limitations 26 (D;E)** | Discuss limitations and their effects on bias, uncertainty, and generalisability. | **Reported** | pp14-15; detailed limitations include sample size, clustering, availability sampling, no-results, reference standard, technical effects, model selection, and lack of external validation. |
| **Usability 27a (D)** | Describe handling of poor-quality or unavailable input data in implementation. | **Reported** | pp5-6 and pp14-15; QC rules, no-result handling, and future automated QC/indeterminate outputs are described. |
| **Usability 27b (D)** | State required user interaction and expertise. | **Partial** | p4 describes trained operators; pp14-16 describe a future pathology-triage workflow, but final user-training requirements remain for prospective development. |
| **Usability 27c (D;E)** | Discuss next research steps for applicability and generalisability. | **Reported** | pp15-16; prospective multicentre external validation, locked pipeline, multiple operators/devices, workflow and cost outcomes. |
