# Supplementary material for: Detection of Cervical Nodal Metastasis in Oral Squamous Cell Carcinoma Using Elastic Scattering Spectroscopy: Ex Vivo Diagnostic Modeling Study
Source: JMIR Cancer. 2026 Oct 13;12:e103960. doi: 10.2196/103960 (PMC13661036; doi:10.2196/103960)
Supplement: Supplementary file 3 [file cancer-v12-e103960-s003.docx]

# B. STARD-AI completed checklist

*Source: Sounderajah V, Guni A, Liu X, et al. The STARD-AI reporting guideline. Nature Medicine. 2025;31:3283-3289. doi:10.1038/s41591-025-03953-8.*

| **Section / item** | **Checklist recommendation** | **Status** | **Reported in manuscript / comment** |
| --- | --- | --- | --- |
| **Title/abstract 1** | Identify the report as an AI-centred diagnostic accuracy study and report at least one accuracy measure in the title or abstract. | **Reported** | p1; title identifies diagnostic modelling and the abstract reports ROC AUC, sensitivity, specificity, predictive values, and accuracy. |
| **Abstract 2** | Provide a structured summary of design, methods, results, and conclusions. | **Reported** | pp1-2; structured abstract. |
| **Introduction 3** | Give the scientific and clinical background, intended use, novelty/established status, and workflow integration of the index test. | **Reported** | pp2-3 and pp14-16; ESS is positioned as an investigational ex vivo pathology-triage adjunct. |
| **Introduction 4** | State study objectives and hypotheses. | **Reported** | p3. |
| **Study design 5** | State whether data collection was planned before or after index and reference testing. | **Reported** | pp3-4; prospective fresh-tissue acquisition during surgery with later retrospective curation and modelling. |
| **Ethics 6** | Report formal ethics approval or justify why it was not required. | **Reported** | Materials and methods—Ethics Approval reports the application and favourable-opinion dates, approving committee and reference, study scope, provisions for annual local organisational governance checks, and the Chief Investigator’s responsibilities. Written informed consent, deidentification, restricted data access, and the periods of tissue collection, data curation, and modelling are described. |
| **Participants 7** | List inclusion and exclusion criteria at participant and data levels, in the order applied. | **Reported** | p4 participant criteria; pp5-6 spectrum/node QC and no-result criteria. |
| **Participants 8** | Describe how potentially eligible participants were identified. | **Reported** | p4; adults with confirmed oral squamous cell carcinoma undergoing neck dissection and with fresh nodes available. |
| **Participants 9** | State setting, location, and dates of participant identification. | **Reported** | pp3-4 and Table 2 (p21). |
| **Participants 10** | State whether the series was consecutive, random, or convenience-based. | **Reported** | p4; pragmatic availability-based sampling is stated, corresponding to a convenience/availability series. |
| **Dataset 11** | Describe the data source and whether it was routine, study-specific, or from an open repository. | **Reported** | pp3-5; study-specific fresh ESS acquisition with routine histopathology, followed by analysis of archived deidentified data. |
| **Dataset 12** | Describe who annotated the dataset, their experience/background, and how annotation was performed. | **Reported** | p5; consultant head-and-neck pathologists assigned routine node-level histopathological diagnoses; selective deeper levels/IHC are described. |
| **Dataset 13** | Report devices used to capture data and software/version used to engineer the index test, including intended use. | **Reported** | pp4-5 and p8/Table S1; ESS hardware and Python/scientific-package versions are specified. |
| **Dataset 14** | Describe acquisition protocols and preprocessing in sufficient detail for replication. | **Reported** | pp4-8 and Table S1 (pp33-34). |
| **Index test 15a** | Describe the index test in sufficient detail for replication. | **Reported** | pp4-8; hardware, calibration, acquisition, QC, features, model, aggregation, and thresholding. |
| **Index test 15b** | Describe development, training, validation, testing, and external evaluation, with sample sizes where applicable. | **Reported** | pp6-8 and Tables 2-6; repeated nested patient-grouped internal validation; no external evaluation. |
| **Index test 15c** | Define and justify positivity thresholds/result categories, distinguishing prespecified from exploratory. | **Reported** | p7 and Table S1; training-only Youden thresholds and the secondary pooled summary threshold are clearly labelled exploratory/descriptive. |
| **Index test 15d** | Identify the intended end user and required expertise. | **Partial** | p4 describes trained acquisition operators; pp14-16 identify future pathology staff as intended users, but final competency requirements remain to be established. |
| **Reference standard 16a** | Describe the reference standard in sufficient detail for replication. | **Reported** | p5; fixation, blocks, H&E sections, deeper levels, IHC, classification, and blinding. |
| **Reference standard 16b** | Explain why the reference standard was chosen. | **Reported** | pp1-3; histopathology is described as the clinical reference standard. |
| **Reference standard 16c** | Define and justify reference-standard positivity categories, distinguishing prespecified from exploratory. | **Reported** | p5; macro-/micrometastasis and isolated-tumour-cell definitions and node classification. |
| **Blinding 17a** | State whether clinical information and reference-standard results were available to index-test performers/readers. | **Reported** | p4 and pp5-6; nodes were selected before final histopathology, and QC was applied without access to the class label. |
| **Blinding 17b** | State whether clinical information and index-test results were available to reference-standard assessors. | **Reported** | p5; pathologists were blinded to ESS spectra and model outputs. |
| **Analysis 18** | Describe methods for estimating or comparing diagnostic accuracy. | **Reported** | pp7-8; accuracy metrics, calibration, clustered bootstrap CIs, thresholding, and exploratory model comparisons. |
| **Analysis 19** | Describe handling of indeterminate index-test or reference-standard results. | **Reported** | pp5-6 and Table 3; six node-level index-test no-results were reported separately and excluded from modelling. |
| **Analysis 20** | Describe handling of missing index-test and reference-standard data. | **Reported** | pp5-6 and Table 3; QC exclusions, no-results, and unavailable clinicopathological fields are described. |
| **Analysis 21** | Report variability analyses, distinguishing prespecified from exploratory. | **Reported** | p8 and p11/Table S3; aggregation, operator, timing, batch, ICC, LOPO, and preprocessing sensitivity analyses. |
| **Analysis 22** | State intended sample size and how it was determined. | **Reported** | p8; complete eligible cohort, no formal calculation. |
| **Analysis 23** | Describe performance-error analysis and algorithmic bias/fairness assessments, if performed. | **Partial** | p11, Table 7, and Table S2 provide detailed error analysis; no formal fairness assessment was undertaken. |
| **Results 24** | Show participant flow in a diagram. | **Reported** | Figure 3 (p28). |
| **Results 25** | Report baseline demographic, clinical, and technical characteristics of training/validation/test sets, as applicable. | **Reported** | p9 and Tables 1-3 (pp20-22); the study used repeated internal resampling rather than fixed independent sets. |
| **Results 26a** | Report disease-severity distribution among those with the target condition. | **Reported** | p5, Table 1, Table 7, and Table S2; nodal burden, micrometastases/macrometastases, deposit sizes, and extranodal extension. |
| **Results 26b** | Report alternative diagnoses among those without the target condition. | **Partial** | p11 and Table S2 characterise reactive/inflammatory causes among false positives; the full negative-node pathology distribution is not tabulated. |
| **Results 27** | Report the interval and interventions between index and reference tests. | **Reported** | pp3-5; ESS was performed on fresh bivalved nodes before fixation and routine histopathology during the same surgical episode. |
| **Results 28** | State whether datasets reflect the target-condition distribution expected in the intended-use population. | **Reported** | p4 and pp14-15; availability-based sampling and prevalence/transportability limitations are explicitly discussed. |
| **Results 29** | For external evaluation, compare the independent dataset with development/validation/test data. | **N/A** | No external independent dataset was evaluated. |
| **Test results 30** | Cross-tabulate index-test results against the reference standard. | **Reported** | p10, Table 5 (p23), and Figure 6 panel C (p31). |
| **Test results 31** | Report accuracy estimates with precision, such as 95% confidence intervals. | **Reported** | pp10-11 and Tables 5-6; patient-clustered or Wilson/bootstrap CIs are reported. |
| **Test results 32** | Report adverse events from the index or reference test. | **N/A** | Ex vivo specimen study; the ESS measurement added no participant-level procedure. |
| **Discussion 33** | Discuss limitations, potential bias, statistical uncertainty, and generalisability. | **Reported** | pp14-15. |
| **Discussion 34** | Discuss implications for practice and the intended clinical role. | **Reported** | pp14-16; investigational pathology-triage adjunct only. |
| **Discussion 35** | Discuss ethical standards and fairness issues associated with the index test. | **Partial** | Ethics is reported on pp3-4; fairness was not formally assessed and generalisability limitations are discussed on pp14-15. |
| **Other 36** | Give registration number and registry name. | **Reported** | p8; the modelling analysis was not prospectively registered. |
| **Other 37** | State where the full protocol can be accessed. | **Partial** | p8 describes the analysis plan and exploratory status; no separate full protocol location is provided. |
| **Other 38** | Report funding/support and funder role. | **Reported** | p16; no financial support. |
| **Other 39** | Report commercial interests, if applicable. | **Reported** | p16; no conflicts declared. |
| **Other 40a** | Describe dataset and code availability and restrictions on reuse/repurposing. | **Reported** | p16; deidentified data, dictionary, and code may be made available subject to governance and consent. |
| **Other 40b** | State whether outputs are stored, auditable, and available for evaluation if required. | **Reported** | Table S1 documents software, seeds, grids, thresholds, and workflow; underlying analytical materials may be requested subject to governance. |
