# Supplementary material for: Detection of Cervical Nodal Metastasis in Oral Squamous Cell Carcinoma Using Elastic Scattering Spectroscopy: Ex Vivo Diagnostic Modeling Study
Source: JMIR Cancer. 2026 Oct 13;12:e103960. doi: 10.2196/103960 (PMC13661036; doi:10.2196/103960)
Supplement: Supplementary file 4 [file cancer-v12-e103960-s004.docx]

# STARD 2015 completed checklist

*Source: Bossuyt PM, Reitsma JB, Bruns DE, et al. STARD 2015. BMJ. 2015;351:h5527. doi:10.1136/bmj.h5527.*

| **Section / item** | **Checklist recommendation** | **Status** | **Reported in manuscript / comment** |
| --- | --- | --- | --- |
| **Title/abstract 1** | Identify the article as a diagnostic accuracy study using at least one accuracy measure. | **Reported** | p1; title and abstract identify diagnostic modelling and report AUC and operating characteristics. |
| **Abstract 2** | Provide a structured summary of design, methods, results, and conclusions. | **Reported** | pp1-2. |
| **Introduction 3** | Describe scientific/clinical background, intended use, and clinical role of the index test. | **Reported** | pp2-3 and pp14-16. |
| **Introduction 4** | State objectives and hypotheses. | **Reported** | p3. |
| **Methods 5** | State whether data collection was planned before or after index and reference testing. | **Reported** | pp3-4; prospective acquisition with later retrospective modelling. |
| **Participants 6** | Describe eligibility criteria. | **Reported** | p4. |
| **Participants 7** | Describe how potentially eligible participants were identified. | **Reported** | p4. |
| **Participants 8** | State setting, location, and dates. | **Reported** | pp3-4 and Table 2 (p21). |
| **Participants 9** | State whether the series was consecutive, random, or convenience-based. | **Reported** | p4; pragmatic availability-based sampling. |
| **Test methods 10a** | Describe the index test in sufficient detail for replication. | **Reported** | pp4-8 and Table S1. |
| **Test methods 10b** | Describe the reference standard in sufficient detail for replication. | **Reported** | p5. |
| **Test methods 11** | Explain the rationale for choosing the reference standard. | **Reported** | pp1-3; histopathology is the reference standard for nodal metastasis. |
| **Test methods 12a** | Define and justify index-test positivity thresholds/categories, distinguishing prespecified from exploratory. | **Reported** | p7 and Table S1. |
| **Test methods 12b** | Define and justify reference-standard positivity categories, distinguishing prespecified from exploratory. | **Reported** | p5. |
| **Test methods 13a** | State information available to index-test performers/readers. | **Reported** | p4 and pp5-6; acquisition preceded final pathology and QC was pathology-blinded. |
| **Test methods 13b** | State information available to reference-standard assessors. | **Reported** | p5; pathologists were blinded to ESS outputs. |
| **Analysis 14** | Describe methods for estimating or comparing diagnostic accuracy. | **Reported** | pp7-8. |
| **Analysis 15** | Describe handling of indeterminate results. | **Reported** | pp5-6 and Table 3; six no-results were reported separately. |
| **Analysis 16** | Describe handling of missing index/reference data. | **Reported** | pp5-6; QC exclusions, no-results, and unavailable archived variables are described. |
| **Analysis 17** | Describe variability analyses, distinguishing prespecified from exploratory. | **Reported** | p8 and p11/Table S3. |
| **Analysis 18** | State intended sample size and how it was determined. | **Reported** | p8; complete available cohort, no formal calculation. |
| **Results 19** | Show participant flow in a diagram. | **Reported** | Figure 3 (p28). |
| **Results 20** | Report baseline demographic and clinical characteristics. | **Reported** | Table 1 (p20). |
| **Results 21a** | Describe disease-severity distribution among participants with the target condition. | **Reported** | Table 1, Table 7, and Table S2; nodal burden, deposit size/class, and extranodal extension. |
| **Results 21b** | Describe alternative diagnoses among participants without the target condition. | **Partial** | p11 and Table S2 report reactive/inflammatory findings among false positives; the full negative-node pathology distribution is not tabulated. |
| **Results 22** | Report time interval and interventions between index test and reference standard. | **Reported** | pp3-5; ESS preceded fixation and routine histopathology during the same surgical episode. |
| **Results 23** | Cross-tabulate index-test and reference-standard results. | **Reported** | p10, Table 5, and Figure 6 panel C. |
| **Results 24** | Report diagnostic accuracy estimates and precision. | **Reported** | pp10-11 and Tables 5-6; 95% CIs reported. |
| **Results 25** | Report adverse events from index or reference tests. | **N/A** | Ex vivo specimen study; no participant-level index-test procedure. |
| **Discussion 26** | Discuss limitations, potential bias, uncertainty, and generalisability. | **Reported** | pp14-15. |
| **Discussion 27** | Discuss implications for practice and the intended role of the index test. | **Reported** | pp14-16. |
| **Other 28** | Provide registration number and registry name. | **Reported** | p8; not prospectively registered. |
| **Other 29** | State where the full protocol can be accessed. | **Partial** | p8 describes the analysis plan and exploratory status; no separate protocol location is given. |
| **Other 30** | Report funding/support and funder role. | **Reported** | p16; no financial support. |
